# Repeated Exposure to Media Violence Is Associated with Diminished Response in an Inhibitory Frontolimbic Network

**DOI:** 10.1371/journal.pone.0001268

**Published:** 2007-12-05

**Authors:** Christopher R. Kelly, Jack Grinband, Joy Hirsch

**Affiliations:** 1 Department of Radiology, Functional MRI Research Center, Columbia University, Neurological Institute, New York, New York, United States of America; 2 Department of Psychology, Columbia University, Neurological Institute, New York, New York, United States of America; 3 Department of Neuroscience, Columbia University, Neurological Institute, New York, New York, United States of America; 4 College of Physicians and Surgeons, Columbia University, Neurological Institute, New York, New York, United States of America; University of Southern California, United States of America

## Abstract

**Background:**

Media depictions of violence, although often claimed to induce viewer aggression, have not been shown to affect the cortical networks that regulate behavior.

**Methodology/Principal Findings:**

Using functional magnetic resonance imaging (fMRI), we found that repeated exposure to violent media, but not to other equally arousing media, led to both diminished response in right lateral orbitofrontal cortex (right ltOFC) and a decrease in right ltOFC-amygdala interaction. Reduced function in this network has been previously associated with decreased control over a variety of behaviors, including reactive aggression. Indeed, we found reduced right ltOFC responses to be characteristic of those subjects that reported greater tendencies toward reactive aggression. Furthermore, the violence-induced reduction in right ltOFC response coincided with increased throughput to behavior planning regions.

**Conclusions:**

These novel findings establish that even short-term exposure to violent media can result in diminished responsiveness of a network associated with behaviors such as reactive aggression.

## Introduction

Depictions of violent acts are common in the mainstream media [1], and both longitudinal [2], [3] and cross-sectional studies [4], [5] have suggested that sustained exposure to these stimuli may result in real-life aggressive behavior [6]. Such exposure, however, has not been shown to influence the cortical networks that regulate behavior, and as a result there is little neuroscientific support and no plausible mechanism for its proposed effect on viewers.

Because violent media have been claimed to increase reactive aggressive tendencies among viewers [7], we examined whether repeated exposure to these stimuli could diminish activation within a frontolimbic network proposed to regulate this behavior. According to several models [8]–[11], the lateral orbitofrontal cortex projects context-relevant information to the amygdala when the latter detects the presence of a threat. In this way ltOFC may, based on social or environmental cues, suppress the initiation of a response cascade that would otherwise lead to reactive aggression. Therefore, we tested whether violent stimuli, and not others of equal arousal, could induce (1) a gradual response attenuation within ltOFC as well as (2) a concurrent decrease in the functional interaction between this region and the amygdala. Finally, we expected a reduction in ltOFC-amygdala interaction to result in (3) potentiation of areas in this network downstream of the amygdala.

Our results indicate that repeated exposure to violent media produces all of these effects, but that none is elicited using equally arousing non-violent stimuli. Furthermore, we found diminished ltOFC responses to be associated with lower thresholds for reactive aggression, consistent with a model in which ltOFC modulates the emergence of this behavior.

## Results

### The video stimuli

To examine the changes in cortical response during repeated contact with media violence, we scanned 14 volunteer subjects while they watched a series of short film clips depicting violent, fearful, or neutral events. The fearful and neutral clips were both non-violent control conditions used to account for the perception of fearful expressions and physical interaction during the violent clips. The violent stimuli depicted acts of physical violence perpetrated by one human on another without mitigating or unrealistic elements; in general, these contained shootings, stabbings, and other kinds of physical assault. The fearful clips contained strong facial expressions of terror without the presence of an explicit aggressor or threat. Finally, the neutral clips depicted non-aggressive physical interactions, such as dances or sports. All of the clips were extracted from mainstream commercial motion pictures. (For details on each clip, see Appendix S1.)

**Figure 1:**
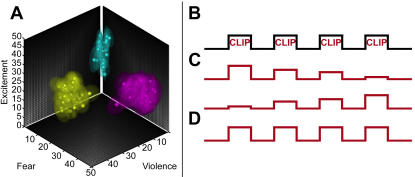
Stimuli and design. (A) Each of the eighty-four clips was rated for violence, fear, and excitement on continuous visual analogue scales (n = 6 subjects). An overall Cronbach's α of 0.88 suggests high inter-rater reliability of stimulus evaluation. Solid spheres represent individual film clips, plotted on each dimension according to their mean rating, while translucent spheres indicate the standard error in each dimension. Spheres are colored according to category (yellow = violent, purple = fearful, blue = neutral). The centers of mass for the three clusters, on (V/F/N) coordinates, are: violent clips [44.4, 28.0, 28.8]; fearful clips [13.5, 43.4, 25.1]; neutral clips [2.5, 3.3, 25.1]. (B) Shown is a schematic of the experimental design, in which the individual film stimuli are presented sequentially with a fifteen second intervening fixation period. (C) To model linear changes in stimulus-evoked response and account for the maximum possible variance, regressors included both a decreasing and increasing function. The regressors were convolved with custom individualized hemodynamic response functions to create the design matrix. Three contrasts were evaluated, one for each regressor and one for their sum (constant activation, [D]). (Note: the actual design contained 24 events.)

There were no significant differences in the average luminance of the clips across the three sets (one-way ANOVA, p>0.05). The qualitative orthogonality of their affective content was verified by a group of additional subjects, who rated the level of violence, fear, or excitement each clip appeared to depict. The summary of these ratings (Figure 1A) demonstrates a clear separation of the stimuli into three distinct clusters on the relevant dimensions. We found no differences in the distribution of the subjective “excitement” ratings among the three sets (two-sided Kolmogorov-Smirnov tests, all p>0.09); however, we also examined differences in attention to each clip by recording the eye movements of ten additional subjects, who watched the stimuli under the same conditions as the scanned subjects. Using repeated measure ANOVAs and post-hoc repeated comparisons (α = 0.05, Bonferroni correction) we examined both total number of saccades and total distance traveled by gaze per stimulus presentation across all subjects and conditions (after normalizing by stimulus lengths), and we found no differences between the violent condition and the control conditions. To determine if prior exposure affected attention to individual clips, we asked subjects to indicate which of the clips they recognized from their own movie-watching experience. Neither eyetracking measure was modulated by prior exposure to individual clips. (See Appendix S2 for more details.)

During each imaging session, subjects passively viewed twenty-four clips (each 2.3 sec±0.38, no audio) from a single category, presented in random order and interleaved with fifteen-second fixation periods (Figure 1B). All subjects thus completed three runs. Because each set of stimuli lasted over seven minutes, all viewing was passive, and the content of many clips was intense, we expected some subjects could become fatigued if all three conditions were presented in sequence. Therefore, each condition was presented on a different day, with the order randomized. Before each run, subjects also viewed a brief, three-minute sequence of natural scenes to minimize the effects of basic visual adaptation.

### Exposure-induced changes in orbitofrontal response

**Figure 2:**
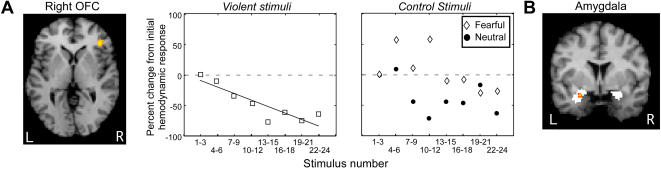
Exposure to violent stimuli diminishes activation of a frontolimbic regulatory circuit. Presented are the significant voxels in right ltOFC for the response attenuation regression analysis, in which the violent condition is compared against the controls. Average hemodynamic responses (HDRs) were extracted on a per-trial basis from this cluster during all three film conditions; these responses were averaged across subjects based on condition type and exposure number, then averaged together into consecutive bins of three. Presented are the maximum signal changes of each binned HDR, expressed as a fraction of the first bin magnitude. The violent clips induced a continuous response attenuation trend that was not present during exposure to the fearful or neutral clips (r^2^_violence_ = 0.79, p = 0.003; r^2^_fearful_ = 0.41, p = 0.09; r^2^_neutral_ = 0.30, p = 0.16). It is important to note that certain areas such as the amygdala were active during the individual conditions alone, or even combined, but that these areas did not show a differential activation between the violent and control conditions. In attention-related areas, all three conditions produced nearly identical attenuation trends; see Fig S2. (B) Psychophysiological interaction analysis identified left amygdala voxels in which functional connectivity with right ltOFC decreased with each exposure to the violent stimuli (p<0.05, mixed-effects t-test). [White underlay is the amygdala ROI, identified using anatomical automatic labeling (AAL).]

We predicted that orbitofrontal regions would show diminished response to each successive violent clip, but remain stable during the fearful or neutral stimuli. A multiple linear regression analysis (Figure 1C–D), which contrasted the three conditions to determine if any frontal regions showed this response pattern, revealed a significant cluster in Brodmann's Area 47/12 of the right hemisphere, defined as the lateral orbitofrontal cortex [12] (right ltOFC; MNI: -42,34,-2; mixed effects t-test, p_resel-corrected_<0.05) (Figure 2A, and see Figure S1 for unthresholded z-statistic image). Hemodynamic responses (HDRs) from this cluster, when averaged across the group, confirmed a progressive decrease in response amplitude to the violent stimuli but not to the fearful or neutral stimuli (Figure 2A).

To confirm that this result did not merely reflect attentional drift specific to the violent condition, we also examined response patterns in the areas most sensitive to changes in overall general attention. To identify these areas, ten additional subjects performed a simple “center-out” saccade task, yielding clusters in middle temporal area (MT), the frontal eye fields (FEF), and the parietal eye fields (PEF) (see methods for experimental details). These clusters were then used as regions of interest to extract average HDRs from the clip-watching runs. These attention-related regions all showed significant and comparable response attenuation patterns for all three conditions, as characterized quantitatively by least squares linear regression. The basic drift-related effect was thus not limited to the violent condition (Figure S2) and therefore could not be responsible for its unique effect on response magnitude over time in right ltOFC. In addition, exposure number had no effect on the amount of saccades made in any of the three conditions (Figure S3), indicating that the attenuation effect in ltOFC could not have resulted from a change in eye movements over time.

To address the possibility that, despite making no overall change in eye movements, subjects were changing their pattern of gaze over time (i.e. looking more or less at the relevant areas of the clip, such as the bodily contact during the violent acts), we drew regions of interest on each frame of each clip. These included the violent acts in the violent clip, the fearful faces in the fearful clips, and the action areas in the neutral clips. We then assessed how often subjects looked within these areas. Subjects consistently spent more than seventy percent of the time looking within the regions of interest (ROIs) for all three conditions (Movie S1 and Figure S4); however, exposure number had no effect on the percent time spent looking in the ROIs for any condition, indicating that the violence-specific attenuation effect in ltOFC could not result from unique changes in gaze pattern over time.

### Exposure-induced changes in orbitofrontal-amygdala interaction

Although it is known that the identified cluster in right ltOFC has extensive connections with the amygdala [13], we hypothesized based on the cited models of reactive aggression that communication between ltOFC and amygdala would be greater during threat (i.e. the violent condition) than baseline. Moreover, given the progressive decline in ltOFC response, we expected this communication to diminish over successive exposures to violent but not control stimuli. The revised psychophysiological interaction (PPI) method [14] was used to model this changing functional correlation with right ltOFC, having removed the main effect of task from the time series data (see methods for additional details). Using a bilateral amygdala mask as a region of interest, we found a significant cluster in the left amygdala (MNI: 26,2,-20; mixed effects t-test, p_resel-corrected_<0.05, Figure 2B). Equivalent analyses of the control stimuli, in contrast, did not produce a significant effect in the amygdala. Functional coupling between ltOFC and amygdala was therefore initially heightened during the violent stimuli but diminished over the course of successive exposures.

### Relevance of these changes to reactive aggression

Exposure to violent media was associated with diminished activation within right ltOFC and reduced right ltOFC-amygdala interaction. To determine whether diminished right ltOFC activation was associated with greater reactive aggressive tendencies, as the OFC-amygdala regulatory model would predict, we correlated right ltOFC response magnitudes with individual assessments of reactive aggressive tendencies, scaled using a modified form of the Buss-Perry aggression questionnaire (BPAQ) [15]. The full BPAQ is a twenty-nine item scale of aggressive inclinations that correlates well with real-life violent behavior [16]. Because it is self-administered, rather than based on clinical evaluations, the scale is well-suited for normal populations. We selected questions most relevant to reactive aggressive behavior, resulting in a seventeen-question scale (BPAQ-RA) containing statements such as “Given enough provocation, I may hit another person.” (Table S1 and Appendix S3).

**Figure 3:**
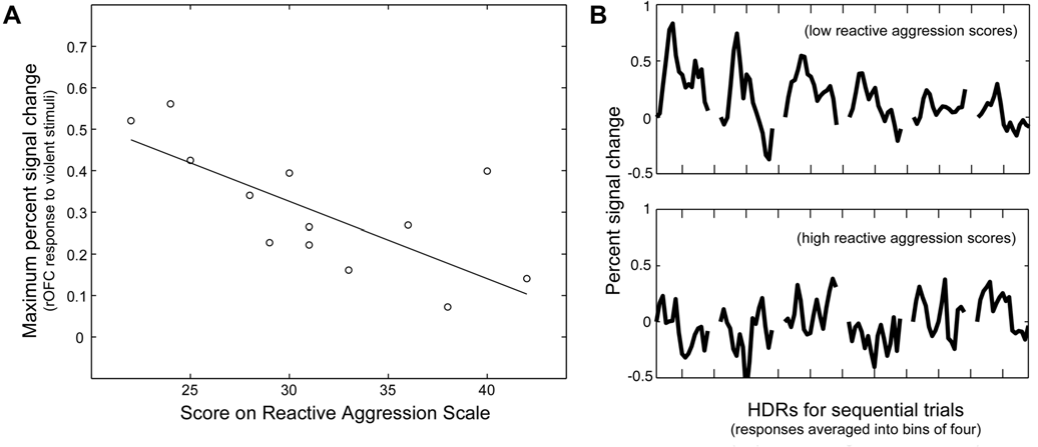
Smaller right ltOFC responses associated with greater reactive aggressive tendencies. (A) Scores on the reactive aggression scale were correlated with the maximum signal change of each subject's average HDR in right ltOFC during the violence exposures (r = −0.69, p = 0.01). Scores spanned a standard, non-clinical range from 22 to 42, out of a possible 85. (Robust-fitted least squares line added.) (B) HDRs (nine-second window) from right ltOFC during the violent condition were extracted from the three subjects with the lowest (top) and highest (bottom) reactive aggression scores and then averaged together based on exposure number. They are presented in sequence, with responses averaged together in bins of four. (The first trace is the average of responses 1–4, the second trace of responses 5–8, and so on.)

There was a significant negative correlation between the peak signal change of each subject's average right ltOFC response during the violent stimuli and his/her BPAQ-RA score (r = −0.69, p = 0.009, Figure 3A); thus, smaller responses in right ltOFC were characteristic of those individuals with greater reactive aggressive tendencies. This trend, however, was unique to the violent condition and not significant for the fearful (r = 0.25, p = 0.41) or neutral (r = 0.07, p = 0.82) conditions, consistent with a model where ltOFC is engaged to regulate reactive aggression in response to threat. Furthermore, there was no correlation between reactive aggression and response in attention-related areas (FEF/PEF/MT, activated during the saccade task) for any condition (all p>0.2). The effect of trait on right ltOFC response was thus not likely related to individual differences in attention. [Two-sided Fisher's tests of correlation differences revealed that, in the right ltOFC, r_violent_ differed from r_fearful_ (p = 0.01) and r_neutral _(p = 0.04); importantly, it also differed from r_violent_ in attention-related areas (p = 0.006).] Finally, to ensure that the correlation between BPAQ-RA scores and right ltOFC response reflected differences in reactive aggression, and not a more general kind of impulsiveness, we asked subjects to complete the Barratt Impulsivity Scale (BIS-11) [17], a standard measure of the latter. BIS-11 scores were not correlated with right ltOFC response (r = -0.35, p = 0.24).

We next correlated subjects' total aggression scores with the peak signal change of their average right ltOFC response magnitudes during early, middle, and late subsets of the violent condition trials to examine the influence of reactive aggressive tendencies on response attenuation within right ltOFC. For the first eight trials, r = −0.60 (p = 0.03); this value dropped to −0.45 (p = 0.12) for trials 9–16 and then to 0.27 (p = 0.37) for trials 17–24 (r_1–8_ and r_17–24_ significantly differ; two-sided Fisher's test of correlation differences, p = 0.03). These data suggest that, among less aggressive subjects, HDRs diminished throughout the violent condition until they became comparable to those of the more aggressive subjects, whose responses were consistently small (Figure S5). To illustrate this point, we averaged per-trial HDRs in the right ltOFC during the violent condition across the three subjects with the highest, as well as the three subjects with the lowest, aggression scores (Figure 3B).

**Figure 4:**
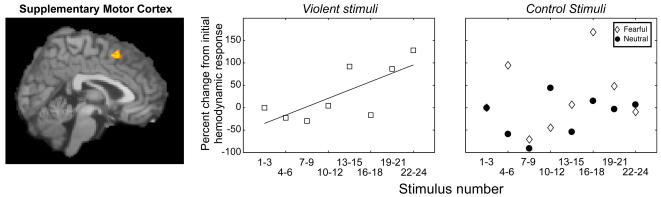
Increased throughput to motor planning regions coincident with diminished regulatory control. The data are analyzed and presented as in figure 2. Exposure to the violent clips induced an increase in supplementary motor response, whereas exposure to the fearful or neutral clips did not (r^2^_violence_ = 0.55, p = 0.03; r^2^_fearful_ = 0.03, p = 0.67; r^2^_neutral_ = 0.12, p = 0.39).

Finally, because of the association between reduced right ltOFC activation and diminished control over the initiation of reactive aggressive behaviors, we tested whether the drop in right ltOFC response would coincide with potentiation of behavior-related regions downstream of the OFC-amygdala interaction–such as, for example, motor planning areas. We therefore examined whether any frontal areas showed *increases* in response during the violent condition but no changes during the control conditions. This analysis yielded a large cluster in the supplementary motor area, a region known to be related to the internal planning and initiation of actions [18] (SMA; MNI: -2,14,52; mixed effects t-test, p_resel-corrected_<0.05, Figures 4 and S6).

## Discussion

Our combined findings show that even short-term exposure to violent media has specific effects on cortical functioning, and that these include diminished activation within a network that regulates behaviors such as reactive aggression.

Although the lateral orbitofrontal cortex has so far been discussed with regard to reactive aggression, there is evidence that this region, especially on the right side, has a more general role in the context-dependent regulation of behavior. Right ltOFC shows robust activation, for example, during tasks of response inhibition where subjects must periodically suppress or change behaviors based on changing external cues [19], [20]. Additional works have shown that these behavior-relevant cues can be social: ltOFC has been observed to become active, for example, when a subject processes second-person accounts of embarrassing actions or violations of social norms [21]. Indeed, those with ltOFC lesions fail to even detect violations of social norms [22], [23]. Right ltOFC has also been shown to be responsive to angry faces [24], which are common indicators that a violation of social norms has occurred [25], and some individuals with ltOFC lesions are unable to detect anger in facial expressions [23]. These combined findings suggest that ltOFC is generally active during situations in which external cues demand that behaviors be changed or suppressed.

The involvement of ltOFC in the regulation of reactive aggression is not surprising if one considers how dependent this behavior is on external contexts. When danger is sensed, for example, one must consider several different factors before deciding how to respond. Is the threat real? Would the environment tolerate aggressive behavior? Is the other individual stronger? Only by integrating these contextual details can one achieve an adequate sense of the situation and respond (or not) accordingly.

In many individuals with ltOFC deficits, the foregoing considerations appear to be either absent or disconnected from the behavioral outcome, with the overall result that minor provocations can trigger inappropriate aggressive responses. In one experiment that divided murderers into predatory and affective groups, affective murderers had less glucose metabolism in lateral prefrontal regions than both predatory murderers and normal controls, who did not differ [26]. Another study found reductions in ltOFC glucose metabolism within subjects that had histories of reactive aggression and impulsive outbursts [27]. A constellation of these ltOFC-related deficits appears in a case report [23] of a patient with damage to this region who, in addition to being hyperaggressive, failed to detect violations of social norms and could not recognize anger in facial expressions. Although each of these symptoms alone could derive from a wide variety of other deficits, their combined presence in this case is consistent with a common involvement of ltOFC.

The above studies indicate that ltOFC integrates contextual information to regulate reactive aggression, as others have proposed [8]–[11]. The data from the aggression questionnaire support this interpretation. Our results, however, extend these findings and indicate that repeated exposure to violent media leads to diminished response in this region and functional disconnection from the amygdala, as well as increased response in motor planning areas.

Although these results are suggestive, further data will be required to assess the specific effects of these functional changes on behavior. Because numerous studies have already linked exposure to violent media with an increase in aggressive behavior [6], it seems reasonable to consider the effect observed here as a plausible component of a mechanism; however, it is important to note that in an otherwise pacific individual, it is very unlikely that these exposure-related changes are a sufficient catalyst for the emergence of criminal aggression. The strongest evidence for this claim is the fact that, although many individuals watch violent media, relatively few go on to commit criminally violent acts.

Aggression is a complex phenomenon, encompassing numerous distinct behaviors that must derive from a wide range of neural networks. Thus, although we examined short-term changes in the cortical response of adults watching violent stimuli, exposure occurring at different frequencies or at different stages of development may induce other cortical changes that affect aggressive behavior. Certainly, the involvement of other networks in the regulation of reactive aggression and other behaviors is well known. As early as the mid-nineteenth century, for example, it was observed that damage to the ventromedial prefrontal cortex could induce “animal passions” in a patient, manifest as a flagrant disregard for social norms and the welfare of others [28]. Additional studies have demonstrated that these frontal deficits may also render one more prone to commit aggressive acts [29]–[32]. Because some studies have suggested that ventromedial areas monitor stimulus-outcome-reward associations [33], behavioral disorders related to lesions in this area have been proposed to reflect a diminished awareness of social rules and frameworks [34]. Lateral lesions, in contrast, appear to cause *violations* of these rules to go unnoticed. In both cases, increases in violent behavior have been reported. Although changes in ventromedial response were not observed in the present work, this should not be taken as evidence that these regions are not involved in these or other exposure-related effects.

The present results indicate that violent media exert a unique effect on a cortical network that is associated with the regulation of reactive aggression and other context-dependent behaviors. This effect may be part of a broad mechanism that can link exposure to violent media with the emergence or increased likelihood of aggressive behavior. Given the complex nature of aggression, however, it should not be taken as the complete mechanism itself. Further studies should determine the role of other aggression-related networks and examine how and when these changes interact with behavioral phenotypes.

## Materials and Methods

### Subjects

Fourteen healthy volunteers (7 men, 7 women) were recruited from the university community. The mean age was 25±4.8 years. Written and oral informed consent were obtained prior to each session in accordance with all institutional guidelines, under protocol AAAA-3690 approved by Team #1 of the Columbia University Medical Center Institutional Review Board.

### Imaging

Subjects were scanned in a 1.5 T twin-speed GE MRI scanner, with twenty-four axial slices covering the full brain acquired with an echo-planar sequence (TR = 2000 ms, TE = 49ms, flip angle = 60°, slice thickness = 5 mm, slice separation = 0 mm, FOV = 200 mm). High-resolution structural images were acquired using the 3D SPGR sequence (124 slices, 256×256, FOV = 200 mm).

During the fMRI experiment, stimuli were presented using an LCD projector (Sanyo PLCXP30) at a resolution of 1280×1024 pixels and a frame rate of 75 Hz. Subjects viewed the images from a supine position in the magnet using mirrors attached to the head coil. Stimuli were generated and responses collected using the Psychophysics Toolbox running under Matlab 5.2 on a G4 Macintosh computer (OS 9.2). All film clips were extracted from DVDs released by major studios, available at a standard video rental outlet.

Once in the scanner, subjects first viewed three minutes of neutral nature scenes to minimize the novelty effects of stimulus presentation. The experimental runs then lasted approximately eight minutes. Stimuli were video clips, each 2.38±0.38 sec long with an interstimulus interval of 15 sec. The presentation order of the stimuli for each condition was randomized for each subject to eliminate any systematic differences in stimulus intensity, both sensory and cognitive. Subjects were scanned in the three experimental conditions on three separate days, with the order of the conditions randomized. (On a few occasions, subjects were scanned on two conditions in a single day because of their limited availability. In these instances, the runs were separated by the eighteen-minute acquisition of a high-resolution SPGR, allowing the subject to rest.)

The attention localizer was a block design consisting of 15 s on/15 s off periods, in which subjects tracked a dot that moved from the center to one of four eccentric positions located at 0°, 90°, 180°, 270°. The dot moved every 750 ms. During the off period the subject maintained fixation at the center. This task identified areas related to eye-movements and shifts in spatial attention. Ten additional subjects from the university community were recruited for this experiment and consented in the same fashion as above.

### Analysis

fMRI data were brain-extracted, motion-corrected, spatially smoothed (Gaussian kernel, FWHM = 5 mm), high-pass filtered (cut-off = 60 sec), and prewhitened using the FSL software suite [35]. An ROI of primary visual cortex was used to extract the time course for each run and a “smart” basis set (FLOBS [35]) was used to estimate the hemodynamic response function (HRF). The HRFs from the neutral, fearful, and violent conditions were then averaged to create a custom HRF for each subject, which was used for all GLM analyses. To avoid circularity, no inferences were made on primary visual cortex. Pre-processed images were then entered into a multiple linear regression analysis with two regressors, modeling linear increases and decreases in activation per exposure number. Contrasts were set for linear decreases, linear increases, and constant levels of activation, with the final contrast modeled as the sum of the decreasing and increasing regressors.

We used two types of group analyses in order to identify voxels that showed more activity in the violent than in the neutral and fearful conditions. First, we performed a fixed-effects, within-subject contrast of V>F & N (that is, a +2 −1 −1 contrast; V = violent, F = fearful, N = neutral) and then included this contrast in a mixed-effects, between-subject group average analysis. In a second independent analysis, we performed a mixed-effects, across-subject tripled t-test (http://www.fmrib.ox.ac.uk/fsl/feat5/detail.html#TripledTwoGroupDifference) to find V>F and V>N, then we intersected these maps.

The results of these two analyses were nearly identical. Because we wanted to show unthresholded activation maps, we included only the results of the first analysis. To confirm that none of the design matrices were rank deficient, we calculated the condition number for each matrix. Condition number values greater than 30 indicate potential problems with colinearity among predictor variables; however, none of the design matrices reached this value (mean = 1.78±0.04). All HDR analyses were performed using MATLAB (The Mathworks).

### Psychophysiological interaction

Using the right ltOFC cluster as a region of interest, a z-score weighted time series was extracted from each subject's functional image and then deconvolved with the HRF. The deconvolved time series was then demeaned, multiplied element-wise by the demeaned decreasing regressor, and then reconvolved with the HRF. The product regressor thus modeled correlation that was increased during stimulus-on periods (over stimulus-off periods) but decreased during each successive exposure. The product regressor, along with the demeaned right ltOFC time series and decreasing regressor, were then entered into the design matrix. The product regressor was orthogonalized with respect to the other two regressors, which were treated as confounds and thus not used in any contrasts. In this way, the analysis ignored the main effect of task. The analysis was restricted to the amygdala.

### Scaling of individual aggression

Subjects completed an interactive, computerized version of the Buss-Perry Aggression Questionnaire after having completed all three functional imaging runs. One subject was no longer available and thus could not complete the survey.

### Psychophysical measurement of stimulus emotional affect

Clips were presented as in the fMRI experiment, except that following the presentation of each clip, three visual analogue scales sequentially appeared on screen. Six naïve subjects (from the same population as the scanned subjects) were instructed to rate how much “Violence,” “Fear,” and “Excitement” was explicitly contained in each stimulus, with the sequence of the three ratings randomized for each trial. This control experiment was not performed during the imaging runs because it would have interfered with the experience of passive viewing. All subjects were consented as above.

### Eyetracking

Ten naïve subjects (from the same population as the scanned subjects) were instructed to watch the clips while we tracked their eye movements using the Avotec Silent Vision System and iViewX v 1.03 software. These subjects watched the stimuli under the same conditions as the scanned subjects. For each stimulus presentation, we measured the subject's total number of saccades and total gaze distance traveled. We then normalized these values based on stimulus duration and entered them into the repeated measures ANOVAs. We also measured on a sample-by-sample basis whether the recorded gaze position fell in the regions of interest drawn on each frame of each clip. All subjects were consented as above.

## Supporting Information

Figure S1 Unthresholded z-statistic map of violence-specific response attenuation. A contrast to determine which voxels showed greater response attenuation during the violent condition than during the fearful or neutral conditions revealed a highly significant cluster in the right orbitofrontal cortex, using the frontal lobe as a region of interest.
(2.94 MB TIF)

Figure S2 Reduction in HDR amplitude in attention-related areas during repeated exposures to film stimuli. HDRs were extracted on a per-trial basis from the average signal in attention-related areas (MT/FEF/PEF, determined using the center-out saccade task) during all three film conditions; these responses were averaged across subjects based on condition type and exposure number, then averaged together into consecutive bins of three. Presented are the maximum signal changes of each binned HDR, expressed as a fraction of the first bin magnitude. All three conditions produce robust habituation trends (r^2^_violence_ = 0.86, p = 0.0007; r^2^_fearful_ = 0.89, p = 0.0005; r^2^_neutral_ = 0.87, p = 0.0007).
(0.64 MB TIF)

Figure S3 Number of saccades does not change over time. Total saccades during each clip were calculated for each subject, normalized by the clip length, and then averaged across subjects based on condition and exposure number. No trend was present during any condition (least squares linear regression, p = 0.74, 0.24, and 0.93, respectively). These data suggest that a change in the number of saccades over time is not a possible explanation for the exposure-related effect in ltOFC response. Of interest is the fact that saccades were greater during both the violent and neutral conditions than during the fearful condition (post-hoc multiple comparisons, Bonferroni correction, p<0.05); this was likely a result of the fact that objects did not move as rapidly around the screen during the fearful clips and, thus, were more likely tracked with smooth pursuit than with saccades. The fact that the total distance traveled by gaze did not differ across the stimuli (see Supplementary Appendix A) supports this interpretation.
(0.26 MB TIF)

Figure S4 Gaze pattern does not change over time. To determine whether subjects fixated more or less on the relevant components of each clip as a function of time, region-of-interest (ROI) boxes were drawn on each frame of each clip. For each exposure, we calculated the fraction of eye-tracking samples that fell within these ROIs; these values were then averaged across subjects based on condition and exposure number (top row). The values in the bottom row have been normalized by ROI size-that is, values for individual exposures were divided by the total fractional screen area that the ROI occupied, since this latter value represents the fraction of randomly distributed gaze positions that the ROI could capture. These data reveal several important points. First, the top row indicates that subjects spent a majority of the time looking within the ROIs during all three stimulus sets (means = 0.80, 0.78, 0.85, respectively, for the violent, fearful, and neutral conditions). After normalization (bottom row), the data show that subjects still consistently looked within the ROIs more than predicted by chance alone. Within these data, no trend was present during any condition (least-squares linear regression, p = 0.21, 0.38, and 0.70, respectively). These data suggest that a change in the pattern of eye movements over time cannot be a possible explanation for the exposure-related effect in ltOFC response. Normalized ROI fixations were greater during the fearful stimuli than the other conditions (ANOVA followed by post-hoc multiple comparisons, Bonferroni correction, p<0.05); this difference, however, most likely reflects the fact that the boxes drawn on the fearful clips were smaller, due to the more constrained space of interest, resulting in larger values after normalization.
(0.48 MB TIF)

Figure S5 Correlation of right ltOFC response magnitude with trait aggression decreases as trials progress. Correlations between signal change in the right ltOFC and individual trait aggression scores were calculated for early (trials 1–8, blue dots) and late (trials 17–24, red dots) onset presentations of the violent stimuli. Responses diminished in subjects with low trait aggression until they were equivalent to those in subjects with higher trait aggression. r_trials 1–8_ = −0.60 [p = 0.032]; r_trials 17–24_ = 0.27 [p = 0.37]. r_trials 1–8_ and r_trials 17–24_ significantly differ; two-sided Fisher's test of correlation differences, p = 0.03.
(0.16 MB TIF)

Figure S6 Unthresholded z-statistic map of violence-specific response potentiation. A contrast to determine if any regions showed greater response potentiation (linear increase) during the violent condition than during the fearful or neutral conditions revealed a highly significant cluster in supplementary motor cortex, using the frontal lobe as a region of interest.
(0.98 MB TIF)

Table S1 This table details the responses of each subject to each question on the modified aggression questionnaire.
(0.10 MB DOC)

Appendix S1 This file contains a description and screenshot of each movie clip.
(1.21 MB PDF)

Appendix S2 This file contains additional information about the eye-tracking analysis.
(0.04 MB PDF)

Appendix S3 This file contains the modified aggression questionnaire.
(0.04 MB PDF)

Movie S1 Consistency of gaze pattern across subjects. This clip shows the eye positions of ten subjects as they watched one of the violent clips. It demonstrates the consistency in gaze pattern across the subjects, as well as the fact that all subjects reliably fixated on the most violent parts of each clip's imagery.
(3.38 MB MOV)
