# Supplementary figures and images for: Repeated Exposure to Media Violence Is Associated with Diminished Response in an Inhibitory Frontolimbic Network
Source: PLoS One. 2007 Dec 5;2(12):e1268. doi: 10.1371/journal.pone.0001268 (PMC2092389; doi:10.1371/journal.pone.0001268)

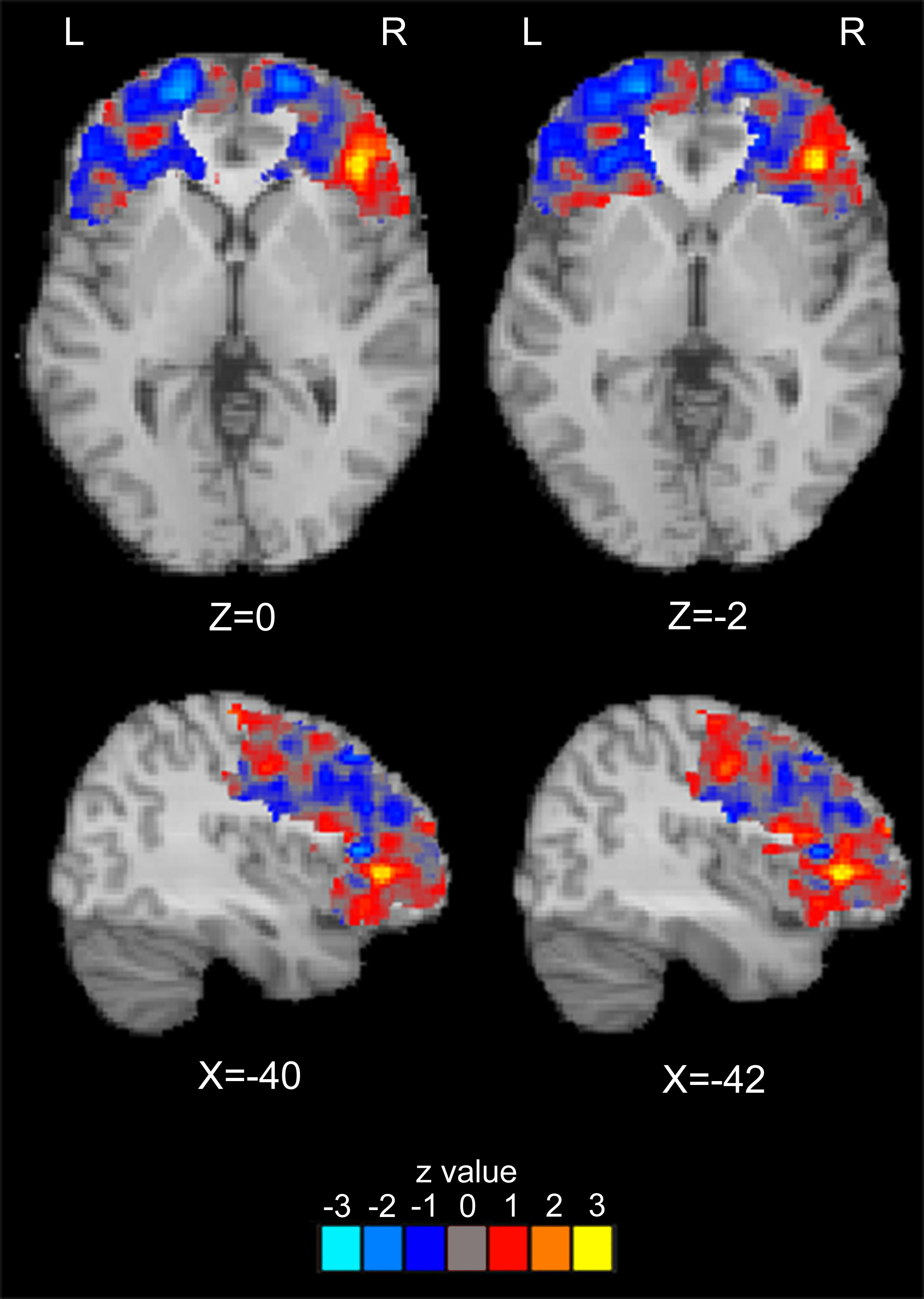

Supplement: Figure S1 — Unthresholded z-statistic map of violence-specific response attenuation. A contrast to determine which voxels showed greater response attenuation during the violent condition than during the fearful or neutral conditions revealed a highly significant cluster in the right orbitofrontal cortex, using the frontal lobe as a region of interest. (2.94 MB TIF) [file pone.0001268.s001.tif]

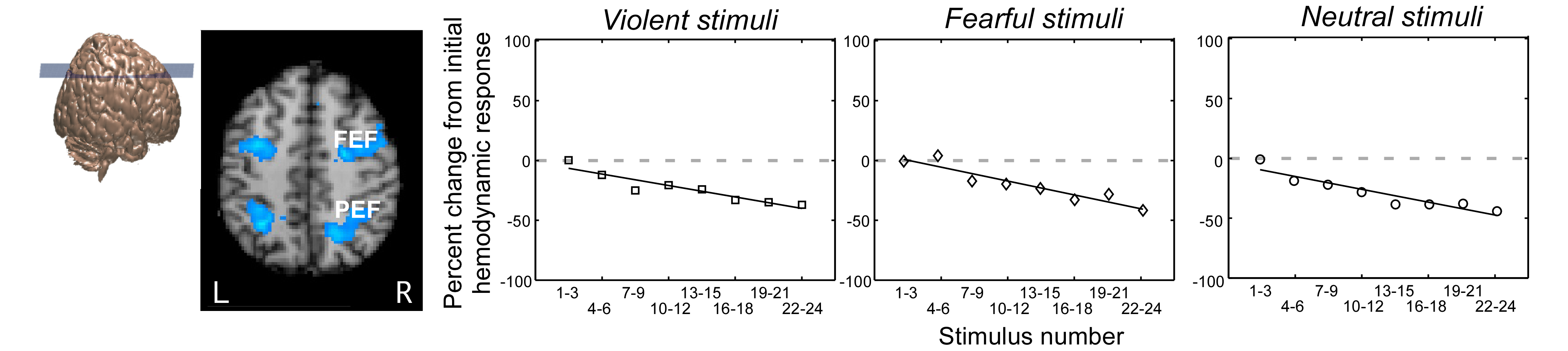

Supplement: Figure S2 — Reduction in HDR amplitude in attention-related areas during repeated exposures to film stimuli. HDRs were extracted on a per-trial basis from the average signal in attention-related areas (MT/FEF/PEF, determined using the center-out saccade task) during all three film conditions; these responses were averaged across subjects based on condition type and exposure number, then averaged together into consecutive bins of three. Presented are the maximum signal changes of each binned HDR, expressed as a fraction of the first bin magnitude. All three conditions produce robust habituation trends (r2 violence = 0.86, p = 0.0007; r2 fearful = 0.89, p = 0.0005; r2 neutral = 0.87, p = 0.0007). (0.64 MB TIF) [file pone.0001268.s002.tif]

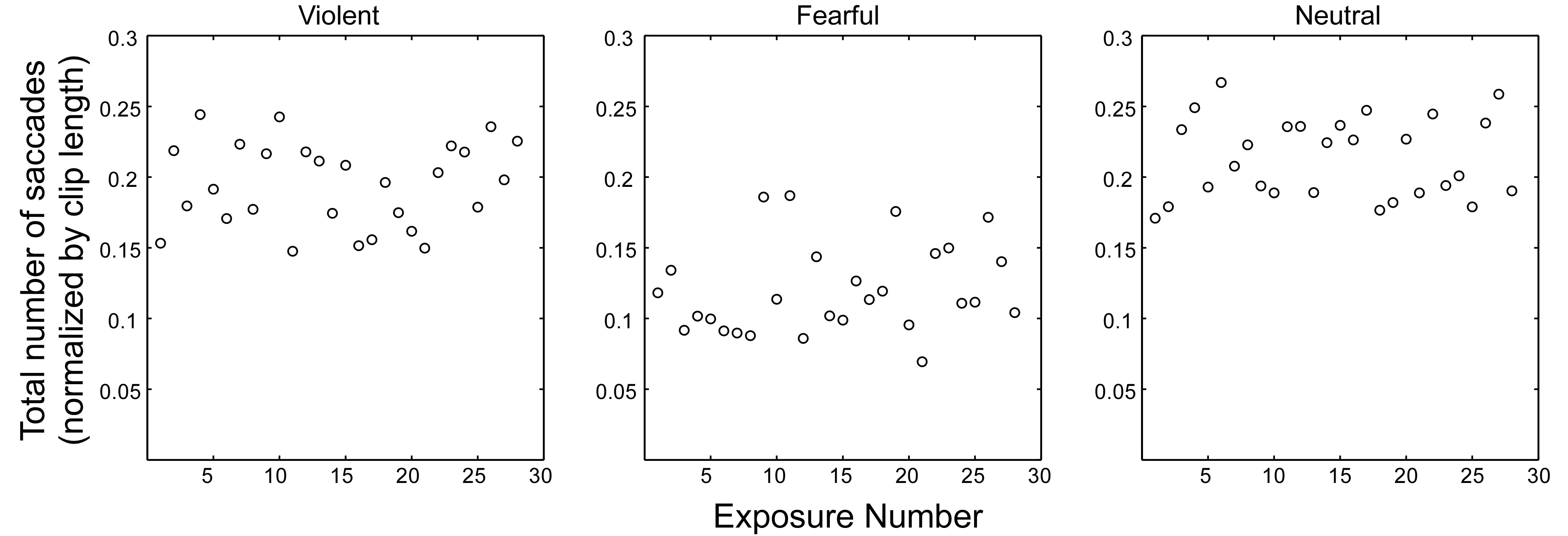

Supplement: Figure S3 — Number of saccades does not change over time. Total saccades during each clip were calculated for each subject, normalized by the clip length, and then averaged across subjects based on condition and exposure number. No trend was present during any condition (least squares linear regression, p = 0.74, 0.24, and 0.93, respectively). These data suggest that a change in the number of saccades over time is not a possible explanation for the exposure-related effect in ltOFC response. Of interest is the fact that saccades were greater during both the violent and neutral conditions than during the fearful condition (post-hoc multiple comparisons, Bonferroni correction, p<0.05); this was likely a result of the fact that objects did not move as rapidly around the screen during the fearful clips and, thus, were more likely tracked with smooth pursuit than with saccades. The fact that the total distance traveled by gaze did not differ across the stimuli (see Supplementary Appendix A) supports this interpretation. (0.26 MB TIF) [file pone.0001268.s003.tif]

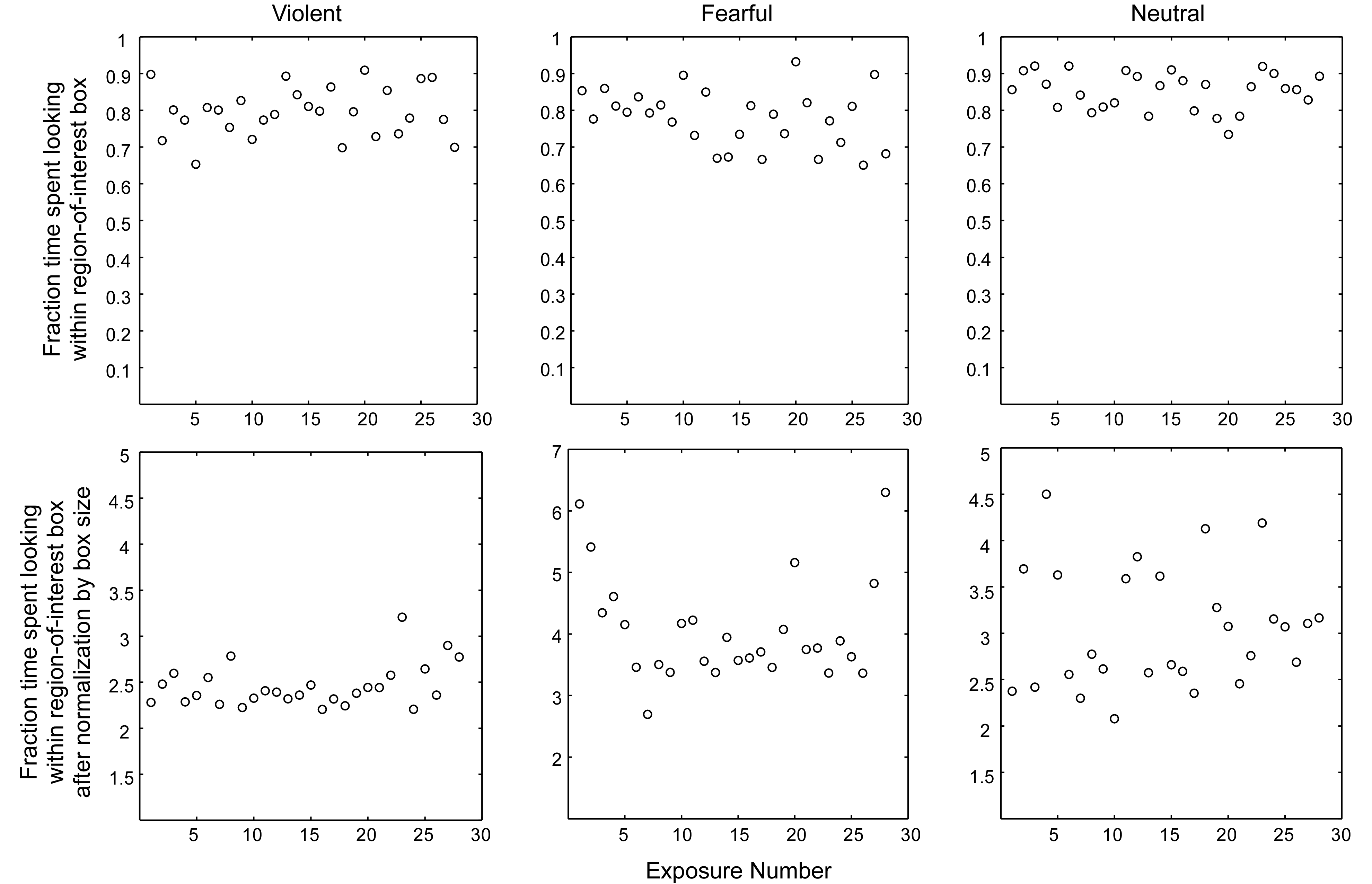

Supplement: Figure S4 — Gaze pattern does not change over time. To determine whether subjects fixated more or less on the relevant components of each clip as a function of time, region-of-interest (ROI) boxes were drawn on each frame of each clip. For each exposure, we calculated the fraction of eye-tracking samples that fell within these ROIs; these values were then averaged across subjects based on condition and exposure number (top row). The values in the bottom row have been normalized by ROI size-that is, values for individual exposures were divided by the total fractional screen area that the ROI occupied, since this latter value represents the fraction of randomly distributed gaze positions that the ROI could capture. These data reveal several important points. First, the top row indicates that subjects spent a majority of the time looking within the ROIs during all three stimulus sets (means = 0.80, 0.78, 0.85, respectively, for the violent, fearful, and neutral conditions). After normalization (bottom row), the data show that subjects still consistently looked within the ROIs more than predicted by chance alone. Within these data, no trend was present during any condition (least-squares linear regression, p = 0.21, 0.38, and 0.70, respectively). These data suggest that a change in the pattern of eye movements over time cannot be a possible explanation for the exposure-related effect in ltOFC response. Normalized ROI fixations were greater during the fearful stimuli than the other conditions (ANOVA followed by post-hoc multiple comparisons, Bonferroni correction, p<0.05); this difference, however, most likely reflects the fact that the boxes drawn on the fearful clips were smaller, due to the more constrained space of interest, resulting in larger values after normalization. (0.48 MB TIF) [file pone.0001268.s004.tif]

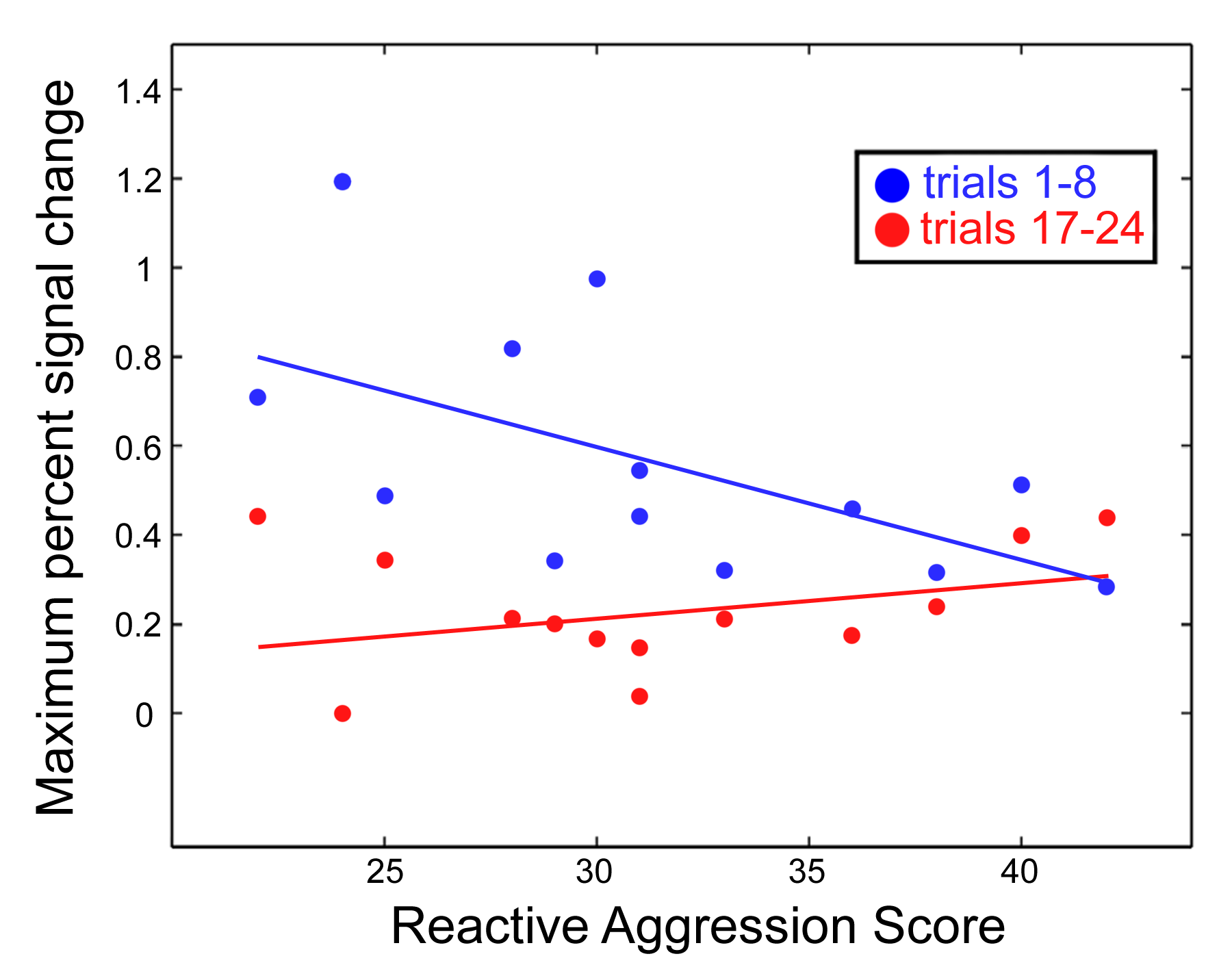

Supplement: Figure S5 — Correlation of right ltOFC response magnitude with trait aggression decreases as trials progress. Correlations between signal change in the right ltOFC and individual trait aggression scores were calculated for early (trials 1–8, blue dots) and late (trials 17–24, red dots) onset presentations of the violent stimuli. Responses diminished in subjects with low trait aggression until they were equivalent to those in subjects with higher trait aggression. rtrials 1–8 = −0.60 [p = 0.032]; rtrials 17–24 = 0.27 [p = 0.37]. rtrials 1–8 and rtrials 17–24 significantly differ; two-sided Fisher's test of correlation differences, p = 0.03. (0.16 MB TIF) [file pone.0001268.s005.tif]

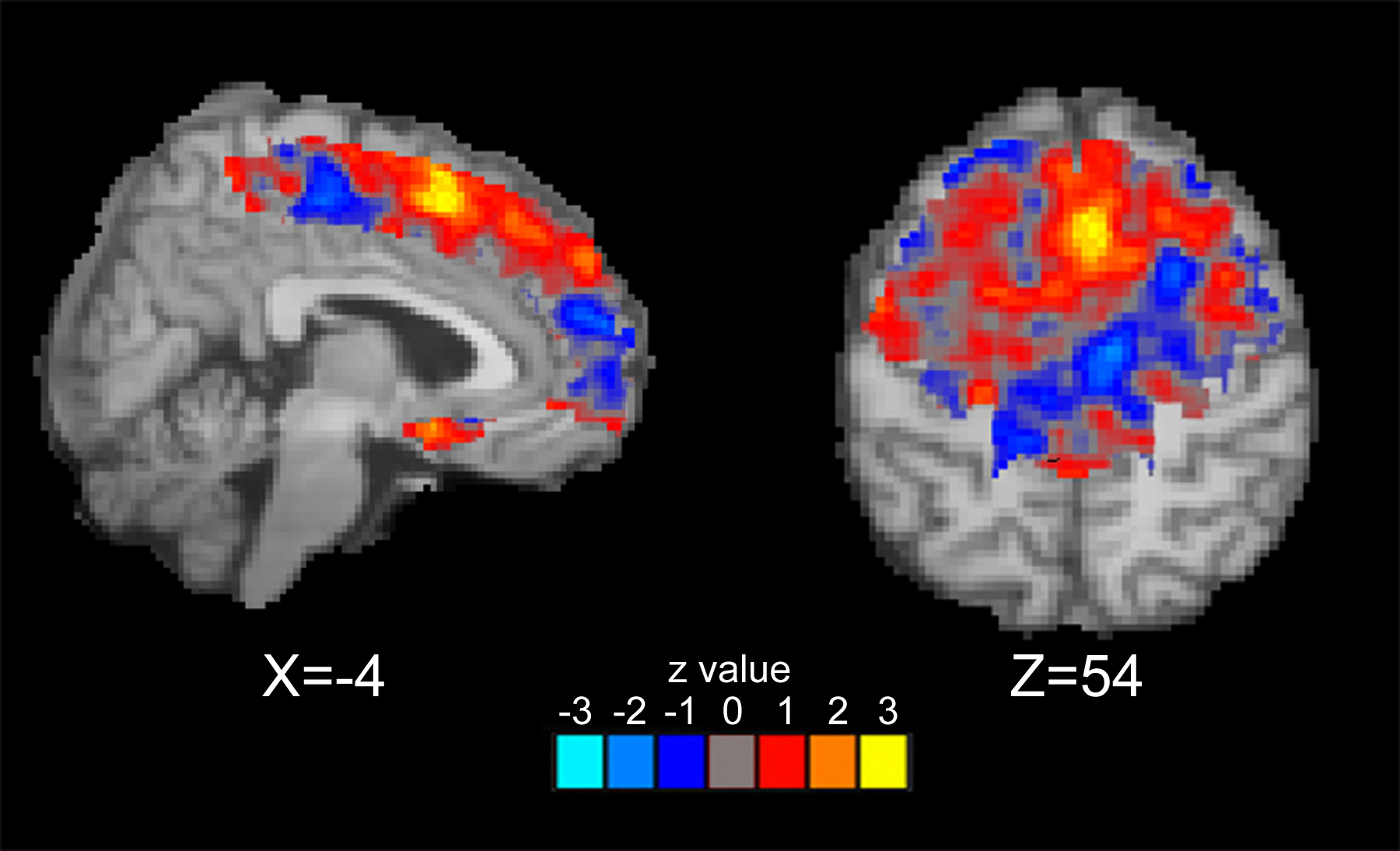

Supplement: Figure S6 — Unthresholded z-statistic map of violence-specific response potentiation. A contrast to determine if any regions showed greater response potentiation (linear increase) during the violent condition than during the fearful or neutral conditions revealed a highly significant cluster in supplementary motor cortex, using the frontal lobe as a region of interest. (0.98 MB TIF) [file pone.0001268.s006.tif]
