# Supplementary material for: Repeated Exposure to Media Violence Is Associated with Diminished Response in an Inhibitory Frontolimbic Network
Source: PLoS One. 2007 Dec 5;2(12):e1268. doi: 10.1371/journal.pone.0001268 (PMC2092389; doi:10.1371/journal.pone.0001268)
Supplement: Table S1 — This table details the responses of each subject to each question on the modified aggression questionnaire. (0.10 MB DOC) [file pone.0001268.s007.doc]

|  | **Question Number** | | | | | | | | | | | | | | | | |  |
| --- | --- | --- | --- | --- | --- | --- | --- | --- | --- | --- | --- | --- | --- | --- | --- | --- | --- | --- |
|  | **1** | **2** | **3** | **4** | **5** | **6** | **7** | **8** | **9** | **10** | **11** | **12** | **13** | **14** | **15** | **16** | **17** | **Total** |
| *Subject 1* | 1 | 5 | 2 | 1 | 2 | 3 | 1 | 4 | 5 | 1 | 4 | 4 | 1 | 1 | 1 | 1 | 1 | 38 |
| *Subject 2* | 5 | 1 | 1 | 1 | 1 | 1 | 1 | 1 | 1 | 1 | 4 | 1 | 1 | 1 | 1 | 1 | 1 | 24 |
| *Subject 3* | 1 | 1 | 1 | 1 | 1 | 1 | 1 | 4 | 2 | 1 | 1 | 2 | 1 | 1 | 1 | 1 | 1 | 22 |
| *Subject 4* | 1 | 4 | 2 | 1 | 2 | 3 | 1 | 3 | 2 | 2 | 4 | 1 | 1 | 1 | 3 | 1 | 1 | 33 |
| *Subject 5* | 1 | 1 | 3 | 1 | 2 | 4 | 1 | 2 | 1 | 3 | 3 | 2 | 1 | 1 | 1 | 2 | 1 | 30 |
| *Subject 6* | 2 | 2 | 2 | 1 | 2 | 1 | 3 | 3 | 5 | 2 | 4 | 5 | 1 | 4 | 3 | 1 | 1 | 42 |
| *Subject 7* | 1 | 1 | 2 | 1 | 2 | 1 | 1 | 2 | 2 | 2 | 2 | 3 | 1 | 1 | 1 | 1 | 1 | 25 |
| *Subject 8* | 1 | 1 | 3 | 1 | 2 | 2 | 3 | 2 | 3 | 1 | 1 | 2 | 1 | 1 | 2 | 1 | 2 | 29 |
| *Subject 9* | 1 | 1 | 2 | 1 | 3 | 4 | 1 | 4 | 1 | 1 | 3 | 1 | 1 | 1 | 3 | 2 | 1 | 31 |
| *Subject 10* | 1 | 1 | 3 | 1 | 1 | 2 | 3 | 3 | 5 | 1 | 2 | 5 | 1 | 3 | 2 | 1 | 1 | 36 |
| *Subject 11* | 3 | 4 | 1 | 1 | 3 | 1 | 1 | 3 | 5 | 3 | 1 | 2 | 5 | 1 | 1 | 4 | 1 | 40 |
| *Subject 12* | 1 | 1 | 1 | 1 | 1 | 1 | 3 | 4 | 3 | 1 | 2 | 3 | 2 | 1 | 1 | 1 | 1 | 28 |
| *Subject 13* | 2 | 1 | 2 | 1 | 2 | 2 | 2 | 3 | 3 | 1 | 3 | 3 | 1 | 1 | 2 | 1 | 1 | 31 |
| Standard Deviation: | 1.19 | 1.46 | 0.76 | 0.00 | 0.69 | 1.15 | 0.95 | 0.95 | 1.61 | 0.78 | 1.19 | 1.39 | 1.12 | 0.96 | 0.85 | 0.87 | 0.28 | 6.17 |

**Supplementary Table 1**

**This table details the responses of each subject to each question on the modified aggression questionnaire.**
