# Supplementary material for: Repeated Exposure to Media Violence Is Associated with Diminished Response in an Inhibitory Frontolimbic Network
Source: PLoS One. 2007 Dec 5;2(12):e1268. doi: 10.1371/journal.pone.0001268 (PMC2092389; doi:10.1371/journal.pone.0001268)
Supplement: Appendix S1 — This file contains a description and screenshot of each movie clip. (1.21 MB PDF) [file pone.0001268.s008.pdf]

Screenshots, credits, and summaries of video stimuli

Violent Clips

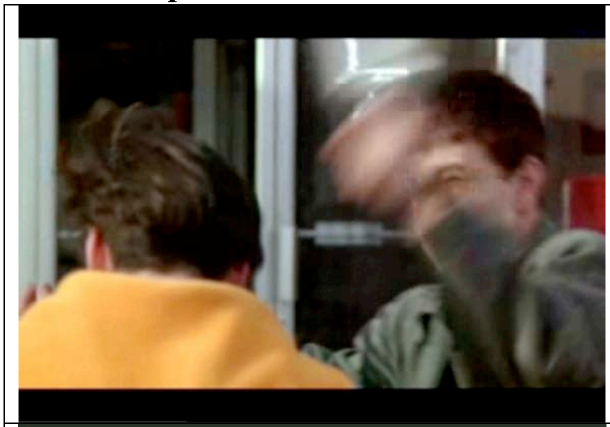	*The Basketball Diaries*. Dir. Scott Kalvert. New Line Cinema: 1995.	A teenager breaks a bottle over another teenager's head.
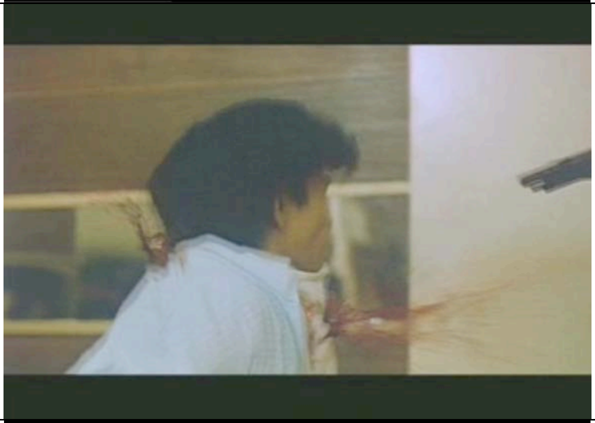	*A Better Tomorrow*. Dir. John Woo. Cinema City: 1986.	One man shoots another at point-blank range in the chest.
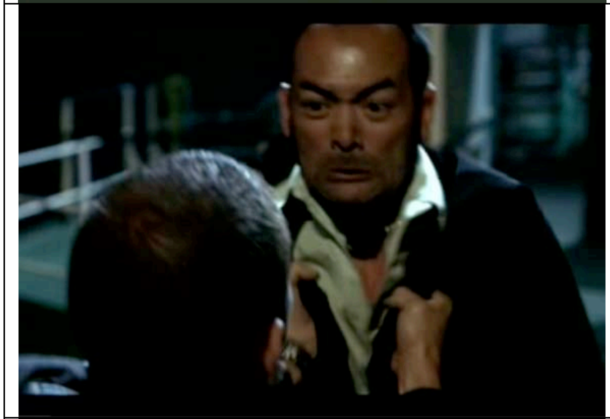	*Wake of Death*. Dir. Philippe Martinez. Bauer Martinez: 2004.	Two men have a fistfight.
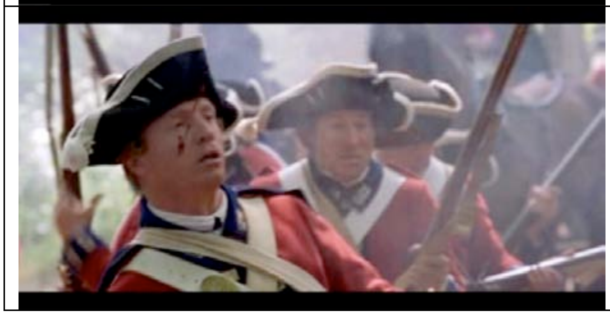	*The Patriot*. Dir. Roland Emmerich. Columbia Pictures: 2000.	A man is shot in the head.

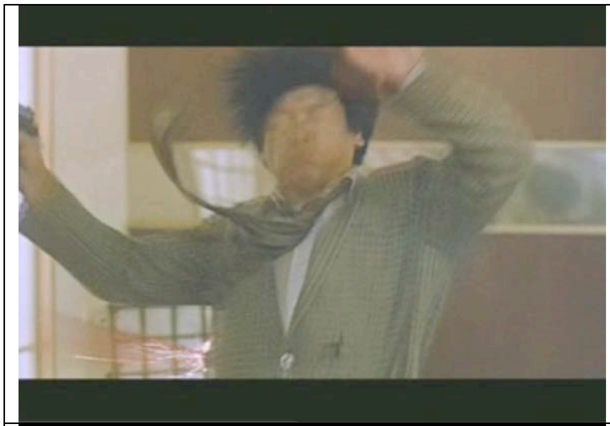	*A Better Tomorrow*. Dir. John Woo. Cinema City: 1986.	One man shoots at another using one gun in each hand.
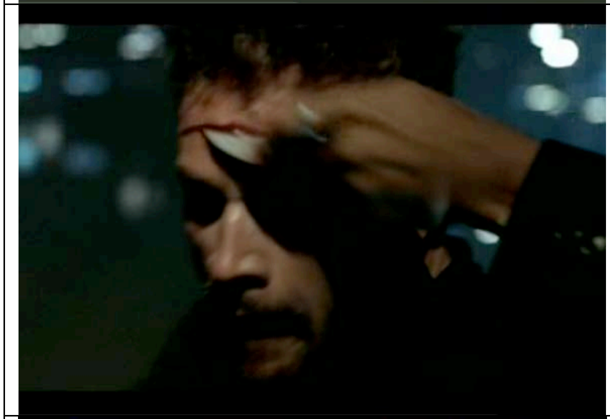	*Blade: Trinity*. Dir. David S. Goyer. New Line Cinema: 2004.	Man reaches around another's face and cuts his forehead with a knife.
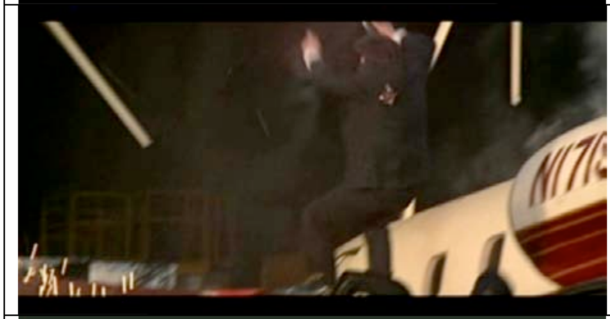	*Face/Off*. Dir. John Woo. Touchstone Pictures: 1997.	Man jumps out of airplane and shoots another man in the chest.
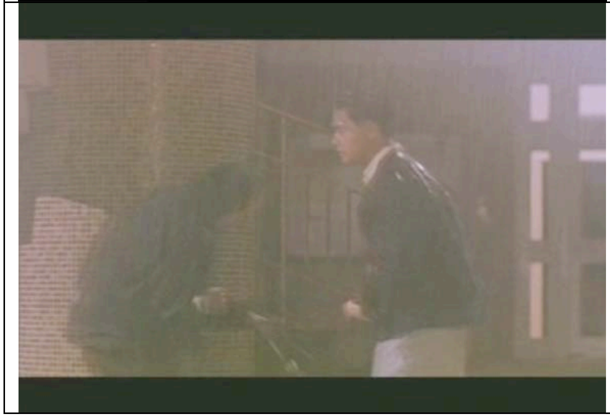	*A Better Tomorrow*. Dir. John Woo. Cinema City: 1986.	Two men have a fistfight in the rain.

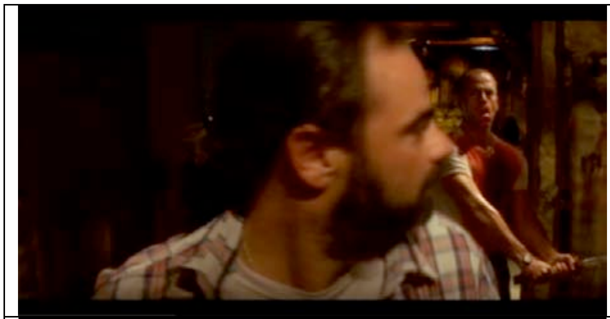	*Pulp Fiction*. Dir. Quentin Tarantino. Miramax: 1994.	One man slashes another in the chest with a sword.
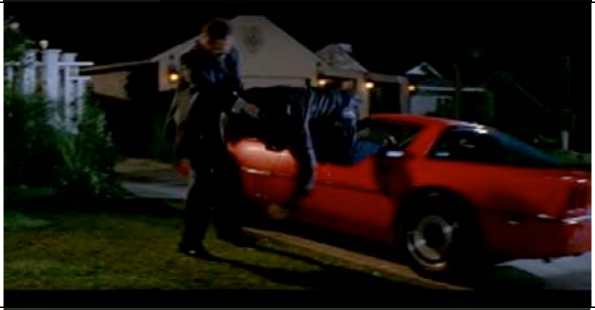	*Face/Off*. Dir. John Woo. Touchstone Pictures: 1997.	Man pulls another out of a car through the window, throws him on the ground.
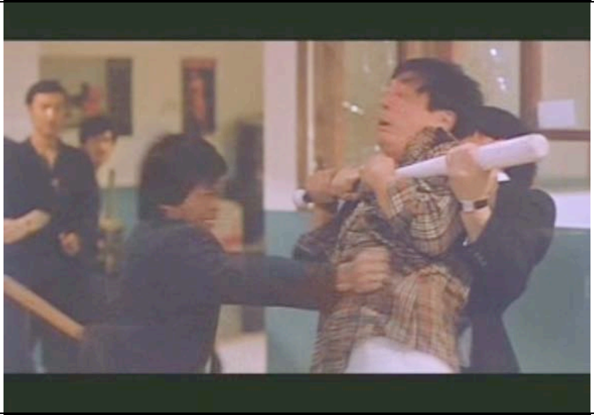	*A Better Tomorrow*. Dir. John Woo. Cinema City: 1986.	Men engage in a mass fistfight; one swings a bat at others.
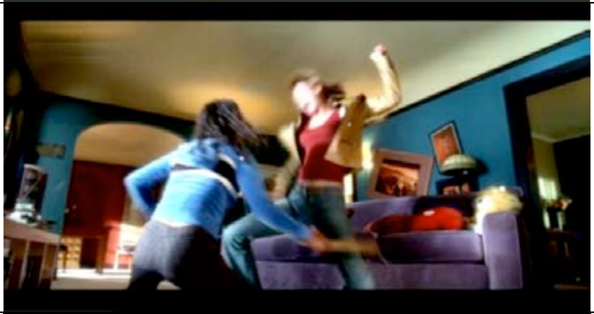	*Kill Bill*. Dir. Quentin Tarantino. Miramax: 2003.	Two women fight; one throws another through a glass table.
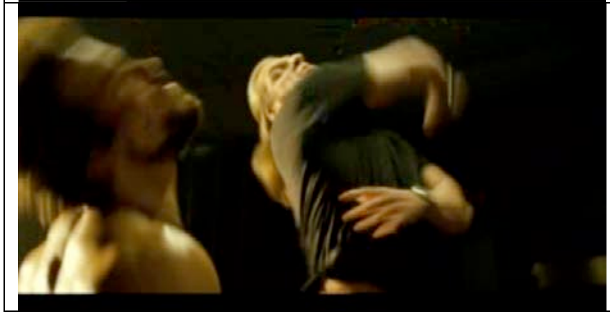	*Blade: Trinity*. Dir. David S. Goyer. New Line Cinema: 2004.	Man delivers a strong uppercut to another.

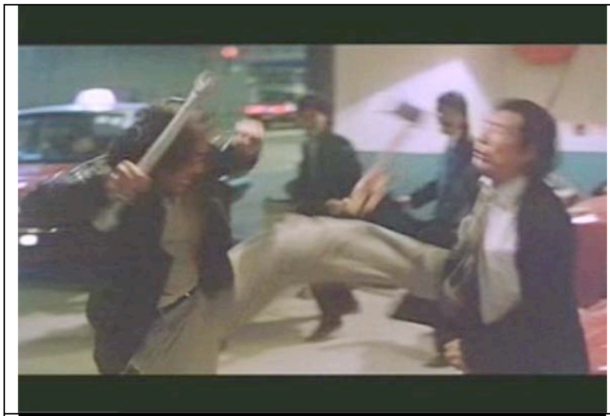	*A Better Tomorrow*. Dir. John Woo. Cinema City: 1986.	Man kicks others and hits them with a wrench.
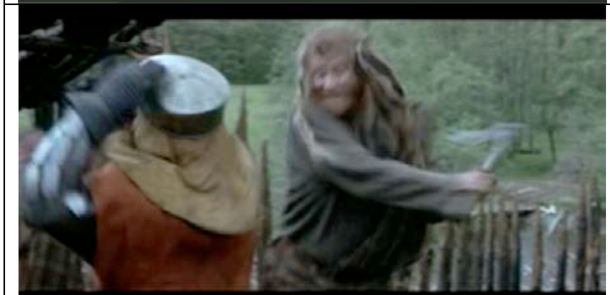	*Braveheart*. Dir. Mel Gibson. 20th Century Fox: 1995.	Man hits another with an axe.
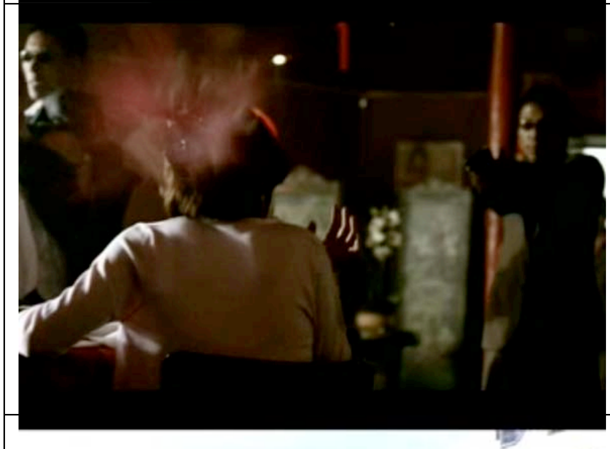	*Wake of Death*. Dir. Philippe Martinez. Bauer Martinez: 2004.	Man shoots woman in the head from point-blank range.
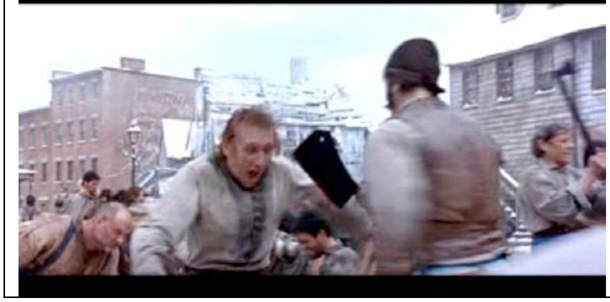	*Gangs of New York*. Dir. Martin Scorsese. Miramax: 2002.	Man strikes another with a meat cleaver.

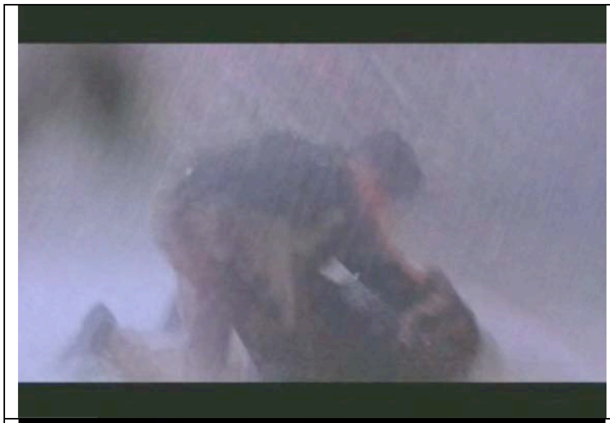	*A Better Tomorrow*. Dir. John Woo. Cinema City: 1986.	Two men engage in a fistfight, wrestle each other to the floor.
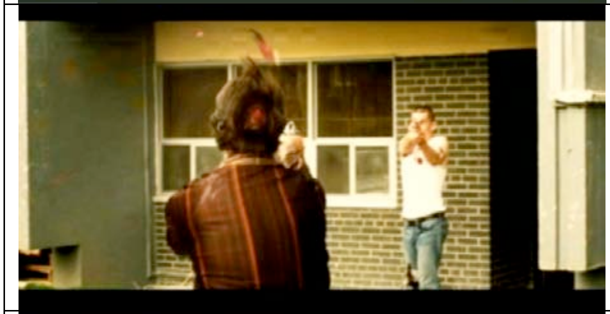	*Assault on Precinct 13*. Dir. Jean-François Richet. Rogue: 2005.	One man shoots another in the head.
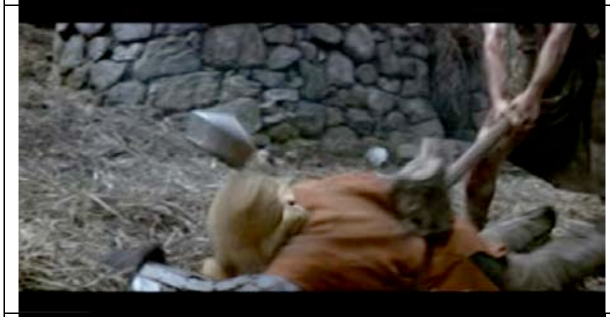	*Braveheart*. Dir. Mel Gibson. 20th Century Fox: 1995.	A man is beaten with a large hammer.
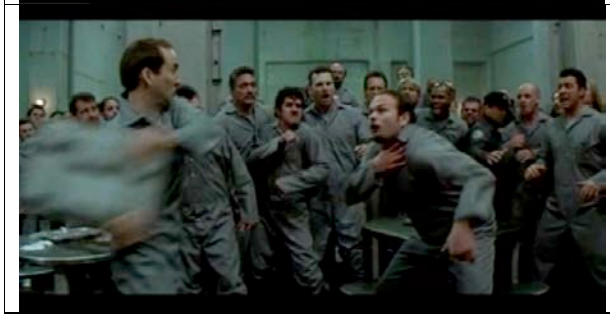	*Face/Off*. Dir. John Woo. Touchstone Pictures: 1997.	A man is hit in the throat and face with a metal tray.

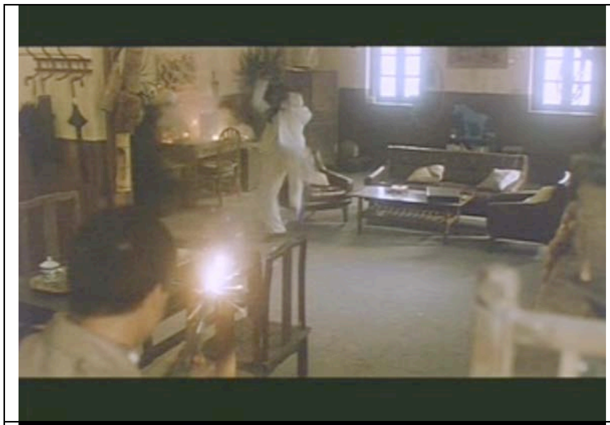	*A Better Tomorrow*. Dir. John Woo. Cinema City: 1986.	Man fires at another with a shotgun, sends victim flying over table.
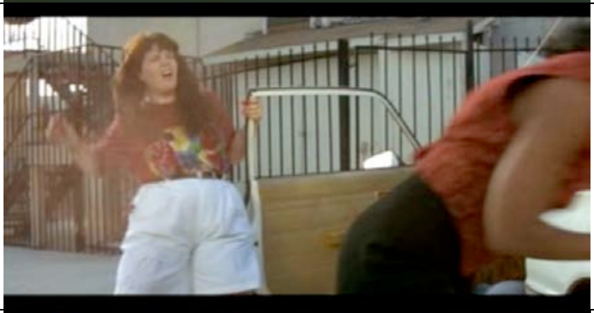	*Pulp Fiction*. Dir. Quentin Tarantino. Miramax: 1994.	Man shoots woman in the leg.
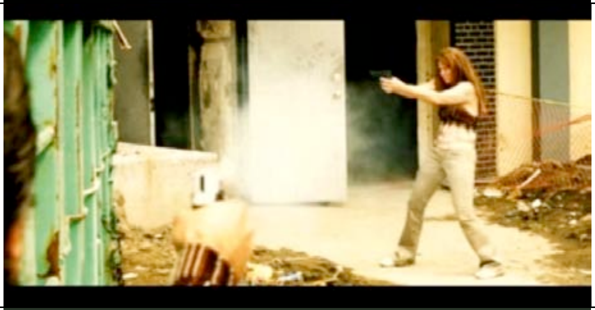	*Assault on Precinct 13*. Dir. Jean-François Richet. Rogue: 2005.	Man shoots woman in the chest.
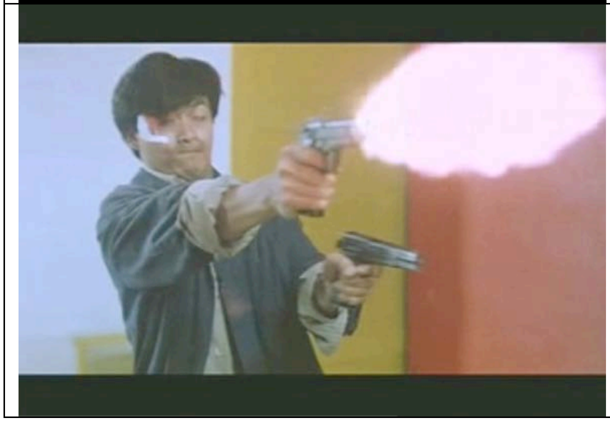	*A Better Tomorrow*. Dir. John Woo. Cinema City: 1986.	Man shoots another in the chest.

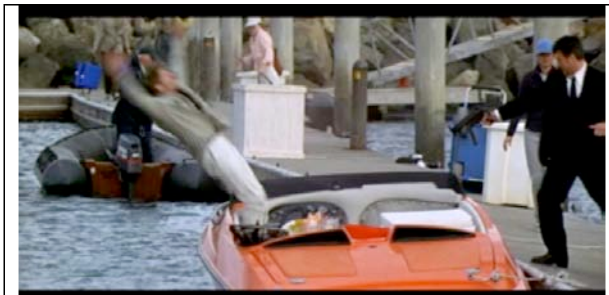	*Face/Off*. Dir. John Woo. Touchstone Pictures: 1997.	Man fires at another with a machine gun, launching him out of boat.
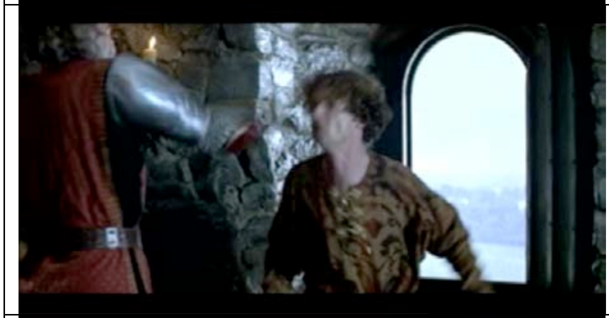	*Braveheart*. Dir. Mel Gibson. 20th Century Fox: 1995.	Man punches younger man in face, knocks him to the floor.
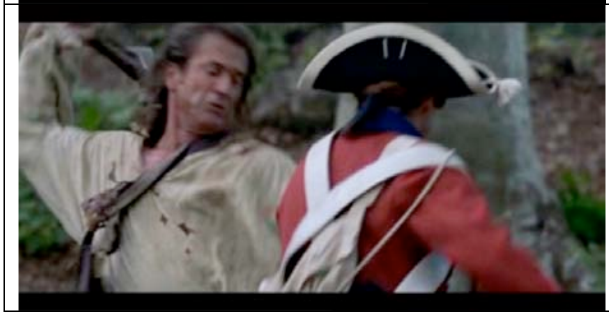	*The Patriot*. Dir. Roland Emmerich. Columbia Pictures: 2000.	Man beats another with an axe.

Fearful Clips

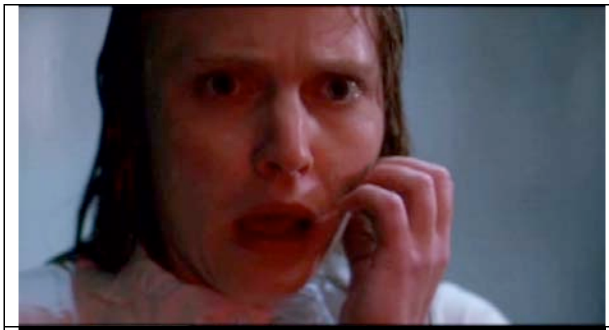	*Nightmare on Elm Street 4*. Dir. Renny Harlin. New Line Cinema: 1988.	Girl expresses dread.
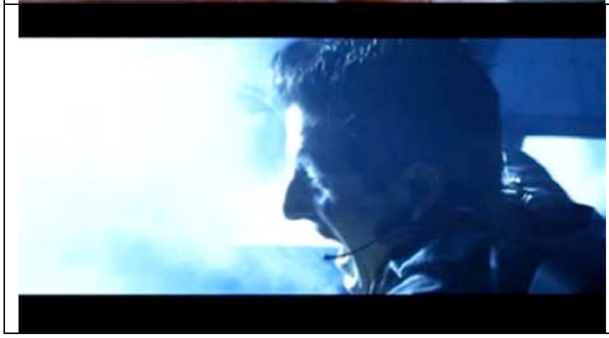	*Armageddon*. Dir. Michael Bay. Touchstone Pictures: 1998.	Two pilots scream and are thrown about in a crash.

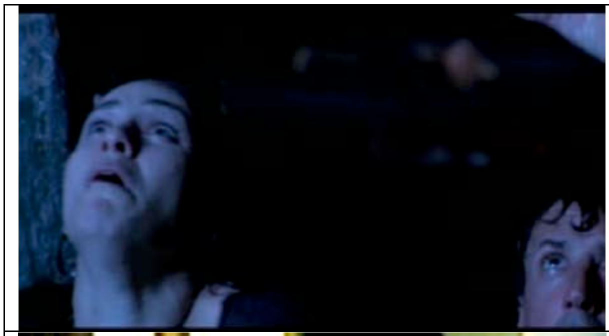	*Daylight*. Dir. Rob Cohen. Universal Pictures: 1996.	Man and woman express fear.
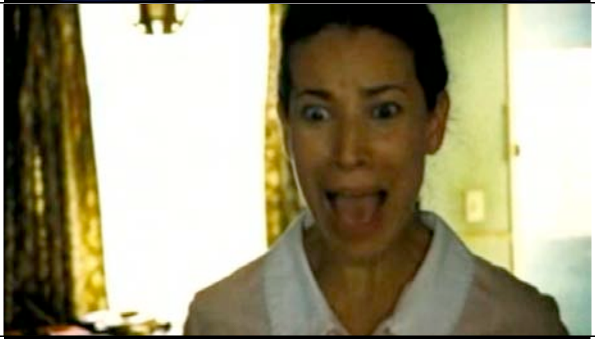	*The Devil's Rejects*. Dir. Rob Zombie. Lions Gate Films: 2005.	Woman screams, as camera zooms in on her face.
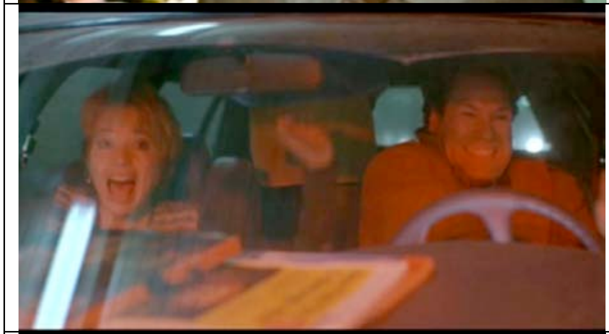	*Daylight*. Dir. Rob Cohen. Universal Pictures: 1996.	Woman screams, man cringes as he swerves his car.
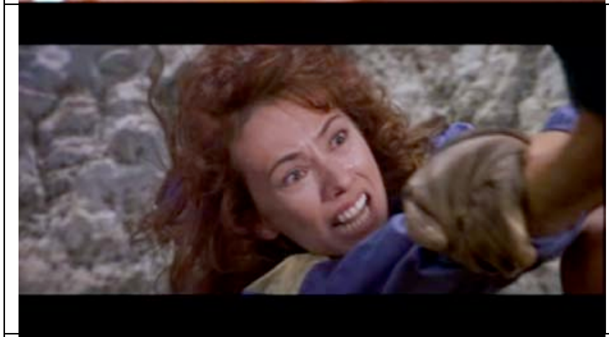	*Cliffhanger*. Dir. Renny Harlin. Canal+: 1993.	Woman expresses fear as she hangs above a canyon.
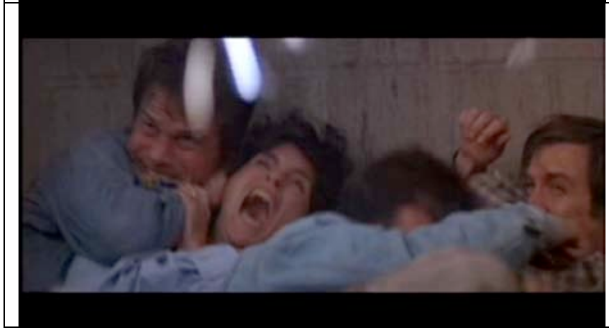	*Twister*. Dir. Jan de Bont. Warner Bros: 1996.	Crowd of people scream, collapse on each other, as sparks fly near them.

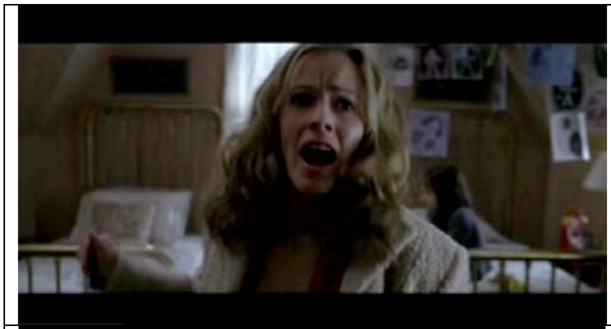	*Hide and Seek*. Dir. John Polson. 20th Century Fox: 2005.	Woman screams, recoils.
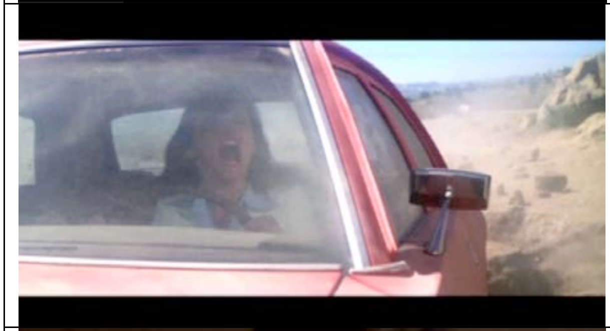	*Superman*. Dir. Richard Donner. Warner Bros: 1978.	Woman screams as her car sinks into a crevice.
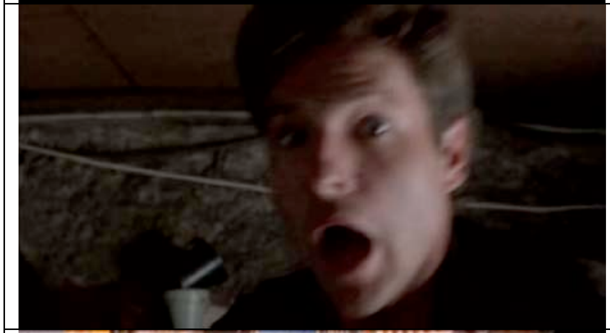	*Pet Semetary*. Dir. Mary Lambert. Paramount Pictures: 1989.	Man shrieks, recoils after a cat jumps out at him unexpectedly.
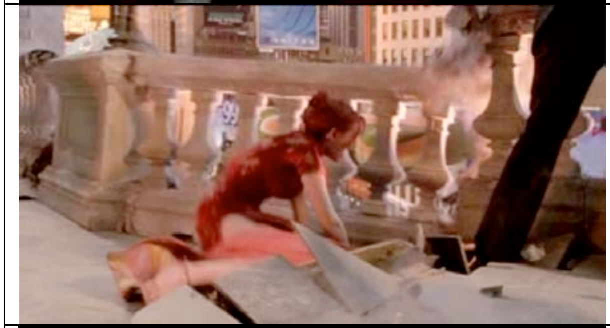	*Spiderman*. Dir. Sam Raimi. Columbia Pictures: 2002.	Woman screams as a balcony collapses beneath her.
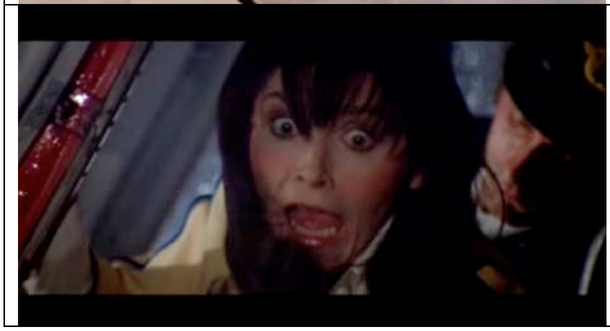	*Superman*. Dir. Richard Donner. Warner Bros: 1978.	Woman screams as she nearly falls out of helicopter.

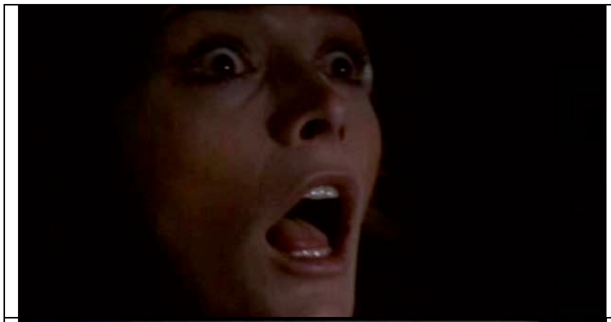	*The Amityville Horror*. Dir. Stuart Rosenberg. MGM: 1979.	Woman appears from darkness and screams.
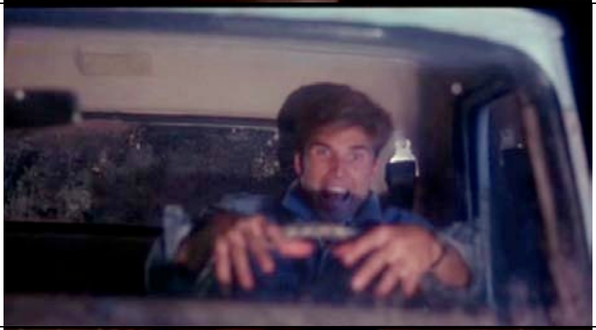	*Nightmare on Elm Street 5*. Dir. Stephen Hopkins. New Line Cinema: 1989.	Man recoils, expresses terror as he is about to be in a car accident.
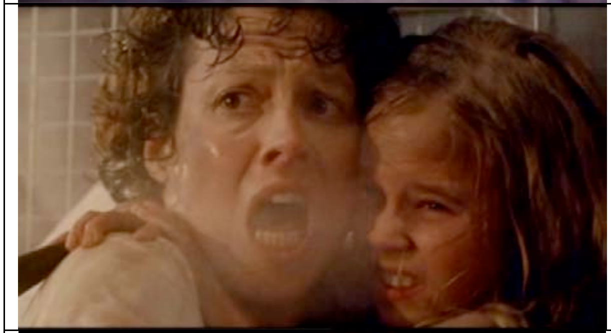	*Aliens*. Dir. James Cameron. 20th Century Fox: 1986.	Woman and girl express horror.
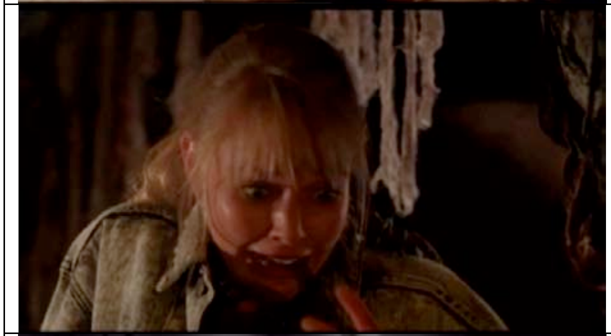	*Nightmare on Elm Street 5*. Dir. Stephen Hopkins. New Line Cinema: 1989.	Girl is terrified when she discovers a tarantula has crawled onto her jacket.
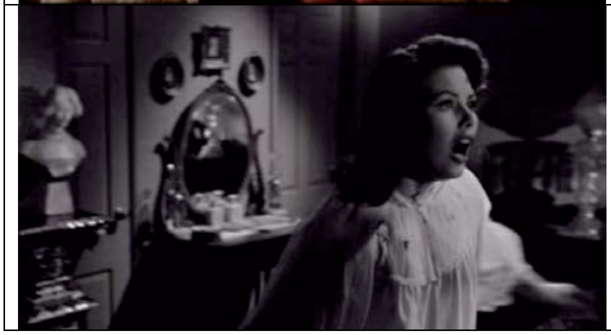	*Thirteen Ghosts*. Dir. William Castle. Columbia: 1960.	Woman backs away with a scared expression on her face.

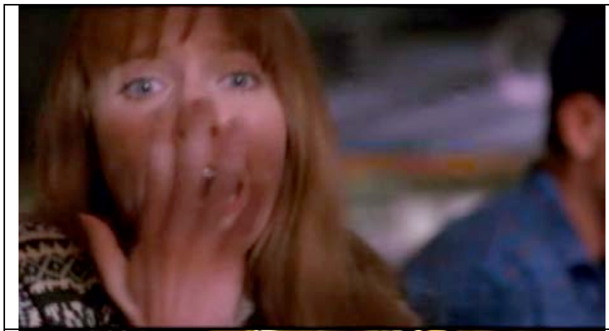	*Nightmare on Elm Street 4*. Dir. Renny Harlin. New Line Cinema: 1988.	Girl starts screaming.
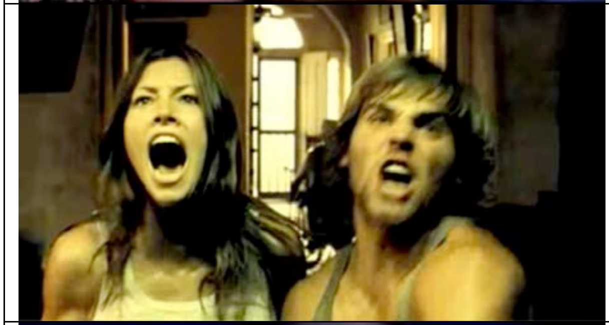	*The Texas Chainsaw Massacre*. Dir. Marcus Nispel. New Line Cinema: 2003.	Young man and woman scream.
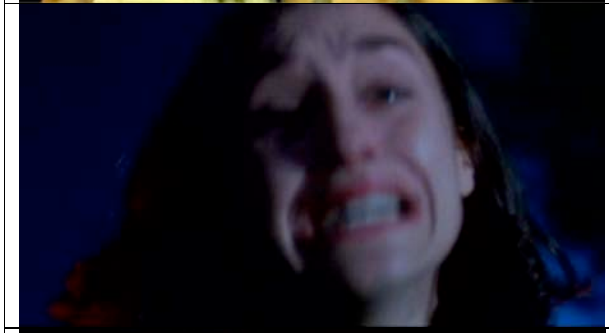	*Freddy's Dead*. Dir. Rachel Talalay. New Line Cinema: 1991.	Girl is hysterically screaming and crying.
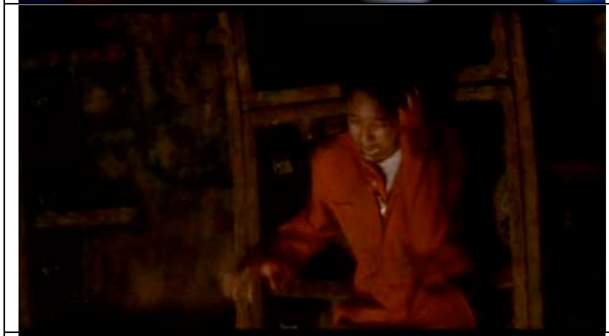	*Daylight*. Dir. Rob Cohen. Universal Pictures: 1996.	Woman is cringing with horrified expression.
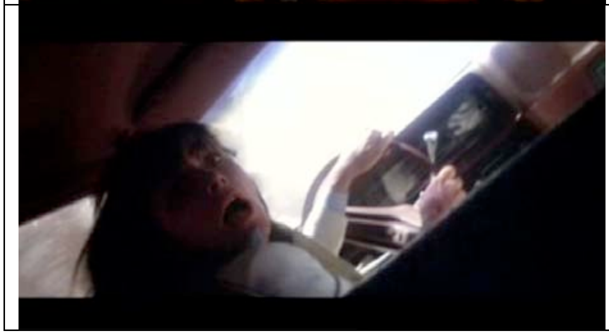	*Superman*. Dir. Richard Donner. Warner Bros: 1978.	Woman is horrified as her car slips into a hole.

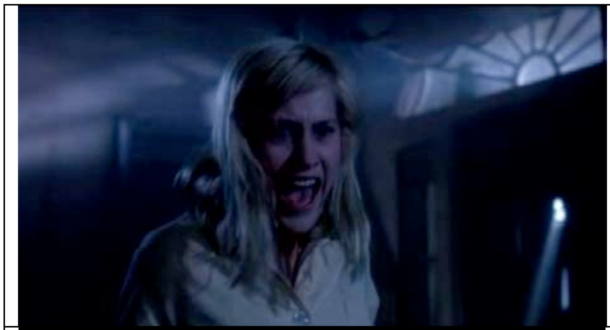	*Nightmare on Elm Street 3*. Dir. Chuck Russell. New Line Cinema: 1987.	Girl screams.
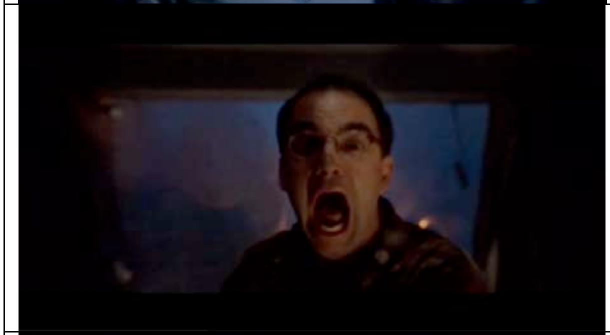	*Godzilla*. Dir. Roland Emmerich. Tristar: 1998.	Man is horrified and screams.
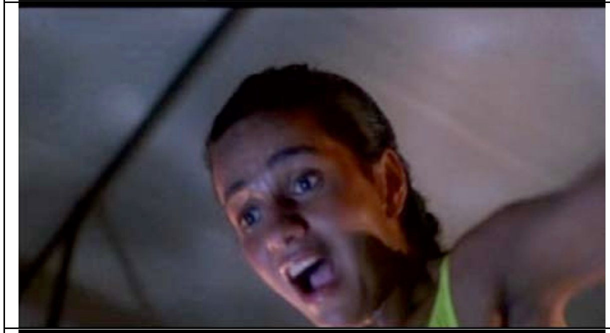	*Nightmare on Elm Street 5*. Dir. Stephen Hopkins. New Line Cinema: 1989.	Girl is slipping at the edge of high dive, shows fearful expression.
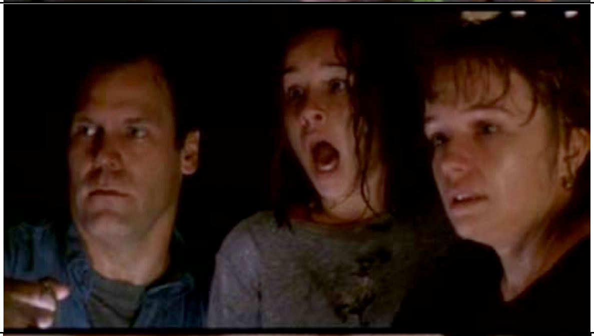	*Daylight*. Dir. Rob Cohen. Universal Pictures: 1996.	Girl and two adults scream.
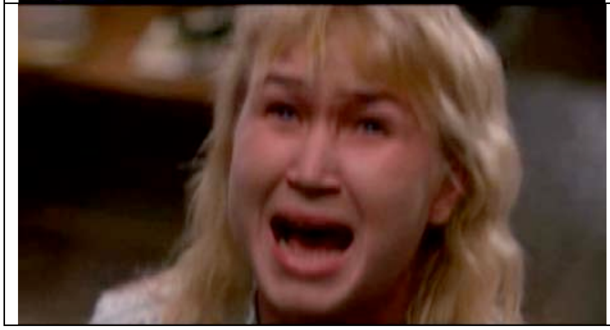	*Nightmare on Elm Street 3*. Dir. Chuck Russell. New Line Cinema: 1987.	Girl cries and screams hysterically.

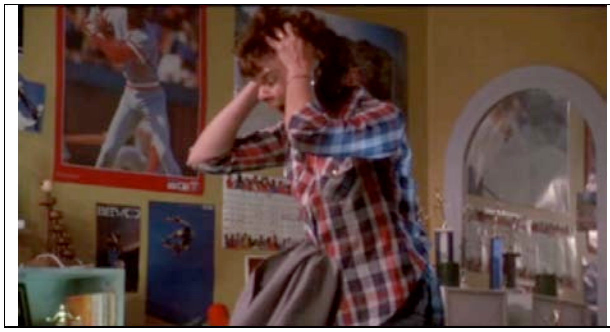	*Nightmare on Elm Street 4*. Dir. Renny Harlin. New Line Cinema: 1988.	Woman pulls back sheets, discovers something, screams in horror.

Neutral Clips

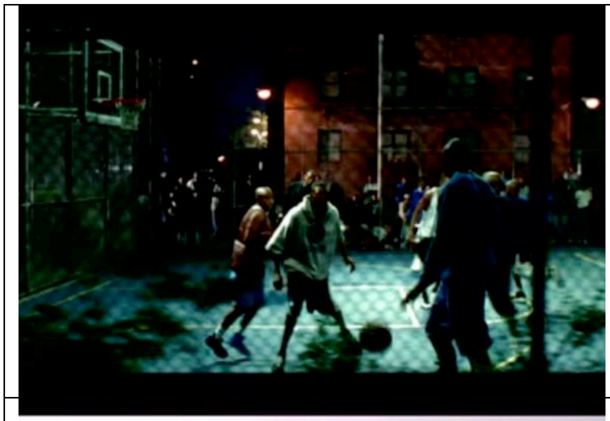	*He Got Game*. Dir. Spike Lee. Touchstone Pictures: 1998.	Men play basketball on a street court at night.
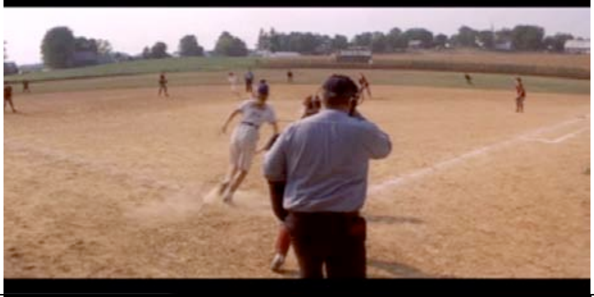	*A League of Their Own*. Dir. Penny Marshall. Columbia Pictures: 1992.	A woman hits a baseball and then starts to run toward first base.
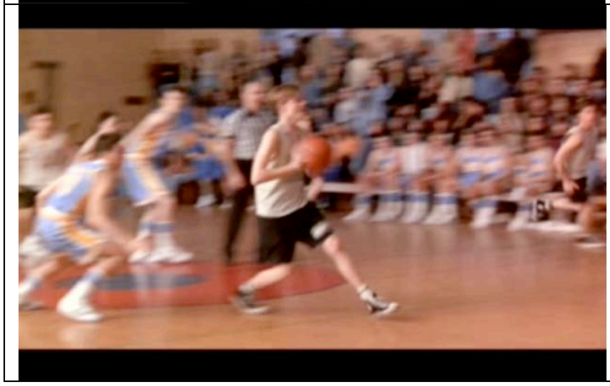	*The Basketball Diaries*. Dir. Scott Kalvert. New Line Cinema: 1995.	A boy throws the ball toward his friend at the basket, who shoots a lay-up.

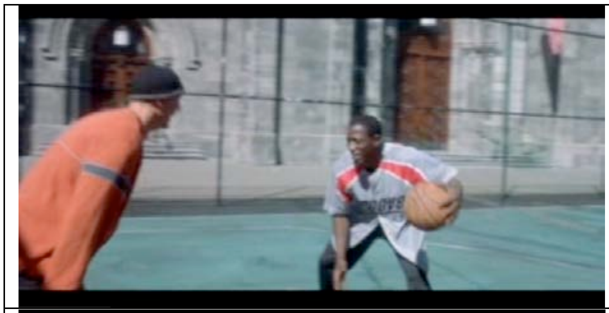	*The Art of War*. Dir. Christian Duguay. Warner Bros: 2000.	Two men play basketball; one tries to block the other as he shoots the ball.
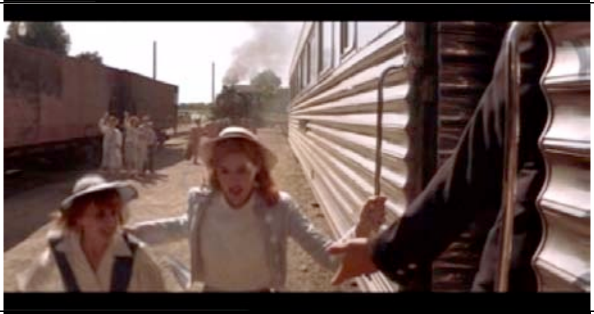	*A League of Their Own*. Dir. Penny Marshall. Columbia Pictures: 1992.	Two women run after a moving train and jump onboard.
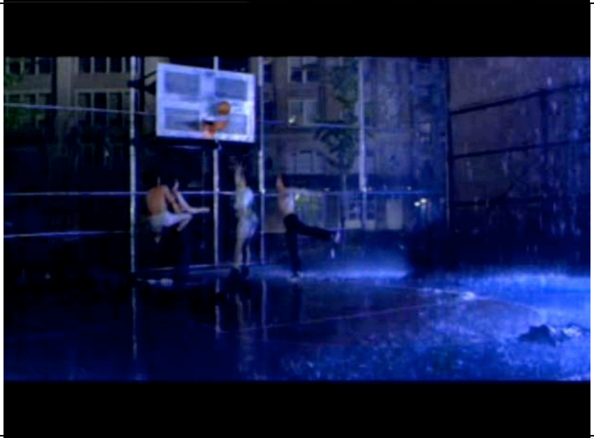	*The Basketball Diaries*. Dir. Scott Kalvert. New Line Cinema: 1995.	Several teenage boys play basketball at night, in the rain.
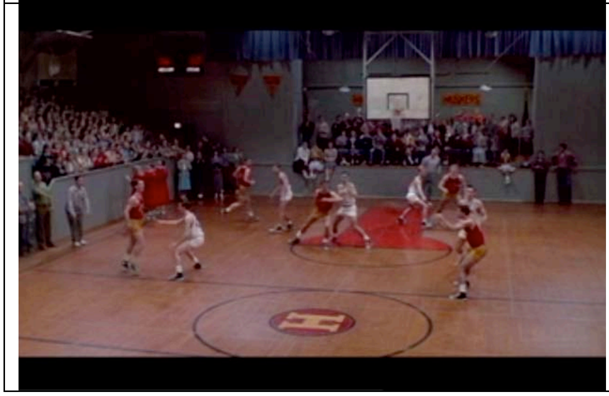	*Hoosiers*. Dir. David Anspaugh. Hemdale: 1986.	Boys pass the ball around while the other team tries to block them.

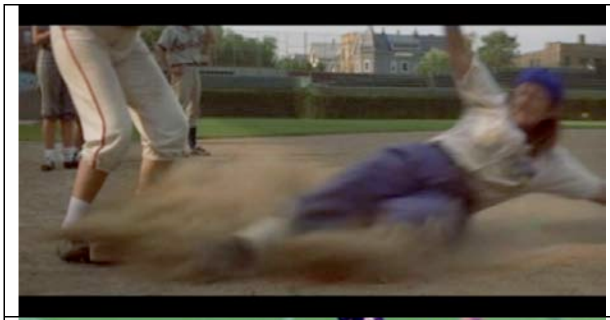	*A League of Their Own*. Dir. Penny Marshall. Columbia Pictures: 1992.	Consecutive shots of women sliding into a baseball plate.
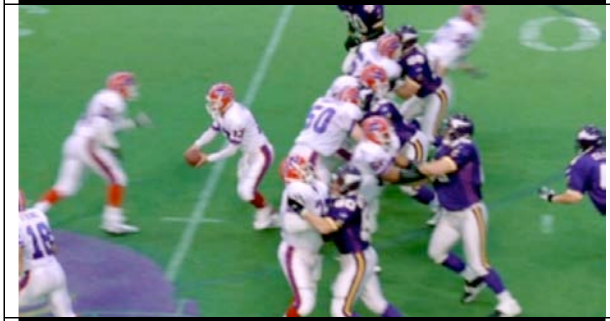	*Second String*. Dir. Robert Lieberman. TNT: 2002.	A player hikes the ball and then hands it off to another, as the opposing team advances.
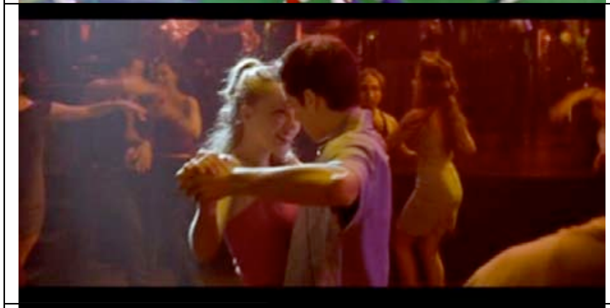	*Center Stage*. Dir. Nicholas Hytner. Columbia Pictures: 2001.	Two young people dance at a nightclub.
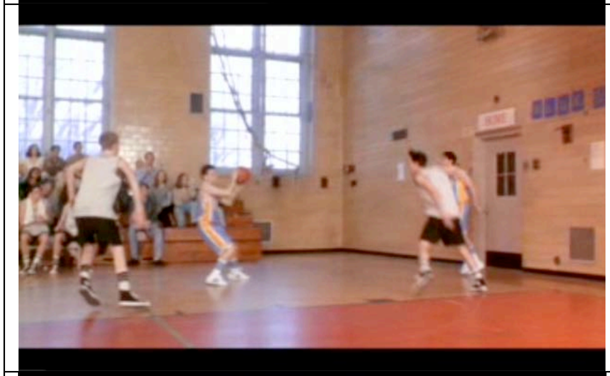	*The Basketball Diaries*. Dir. Scott Kalvert. New Line Cinema: 1995.	Young boys pass a basketball to each other on an indoor court.
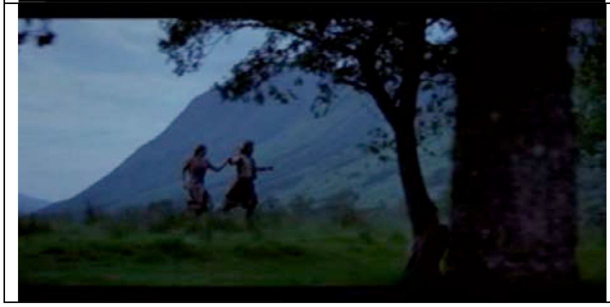	*Braveheart*. Dir. Mel Gibson. 20th Century Fox: 1995.	A man and woman run through the woods together.

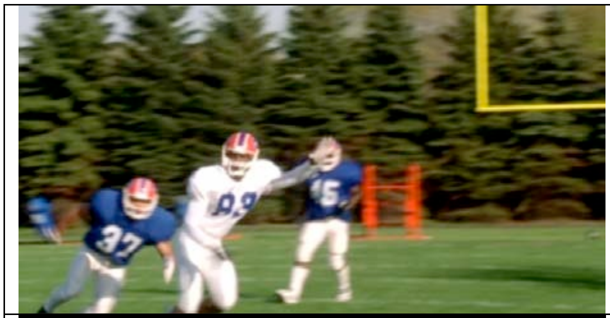	*Second String*. Dir. Robert Lieberman. TNT: 2002.	A player tries to open himself up for a pass.
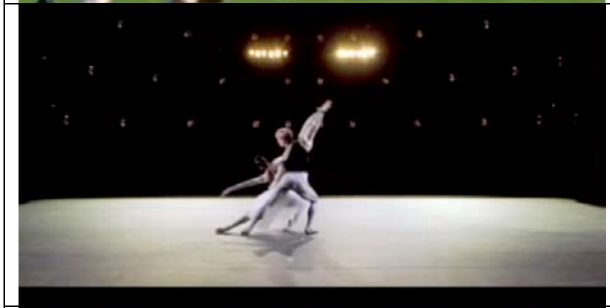	*Center Stage*. Dir. Nicholas Hytner. Columbia Pictures: 2001.	Two people dance ballet on a stage.
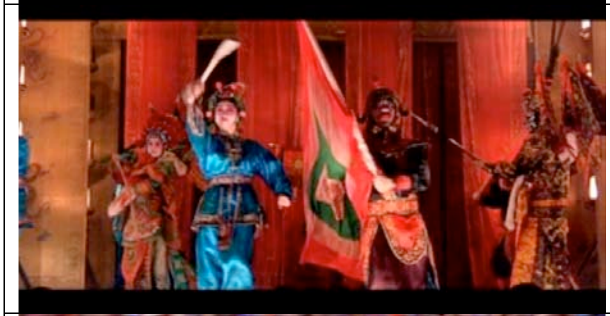	*Gangs of New York*. Dir. Martin Scorsese. Miramax: 2002.	Several people perform a brief dance with flags and swords.
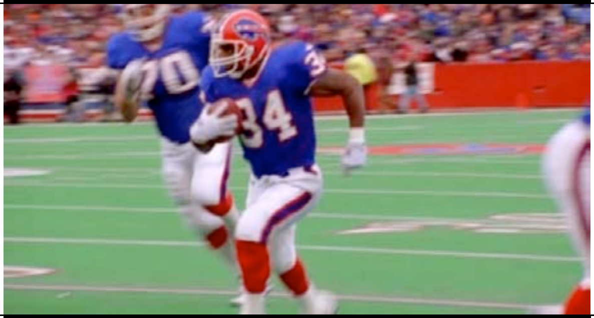	*Second String*. Dir. Robert Lieberman. TNT: 2002.	A man runs with a football, dodging players from the other team.
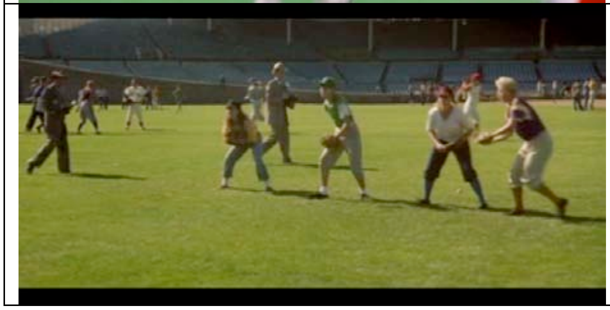	*A League of Their Own*. Dir. Penny Marshall. Columbia Pictures: 1992.	A line of women plays catch with a baseball.

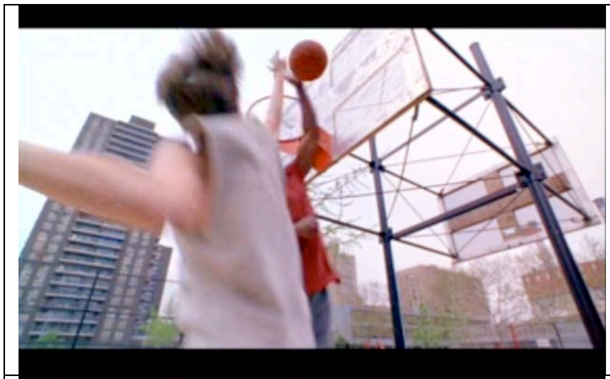	*The Basketball Diaries*. Dir. Scott Kalvert. New Line Cinema: 1995.	A boy shoots for the basket but is blocked by another man.
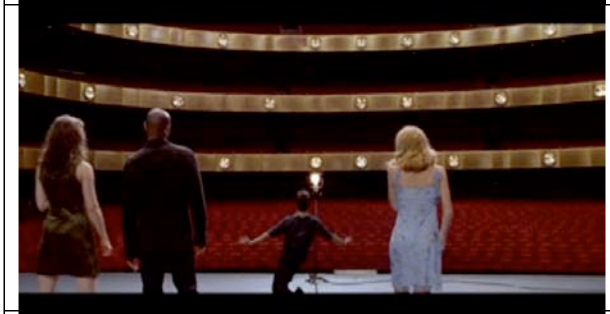	*Center Stage*. Dir. Nicholas Hytner. Columbia Pictures: 2001.	A man dances and kneels in front of an empty auditorium.
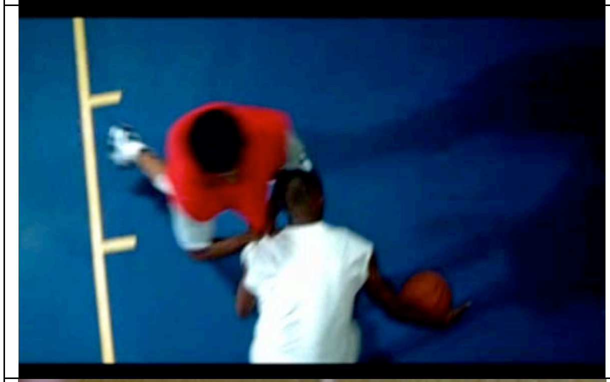	*He Got Game*. Dir. Spike Lee. Touchstone Pictures: 1998.	An aerial shot shows a man trying to advance toward the basket while another blocks him.
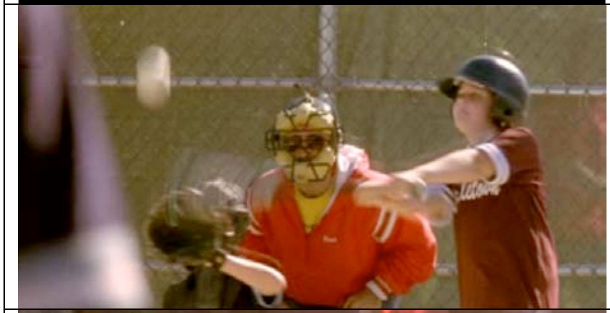	*Malevolence*. Dir. Stevan Mena. Magnetic Media: 2004.	A girl swings and misses the baseball.
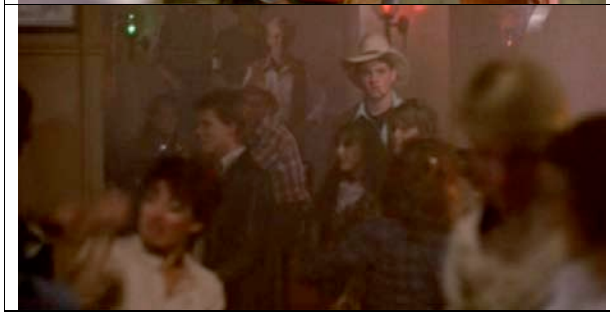	*Footloose*. Dir. Herbert Ross. Paramount: 1984,	A crowd of people dance.

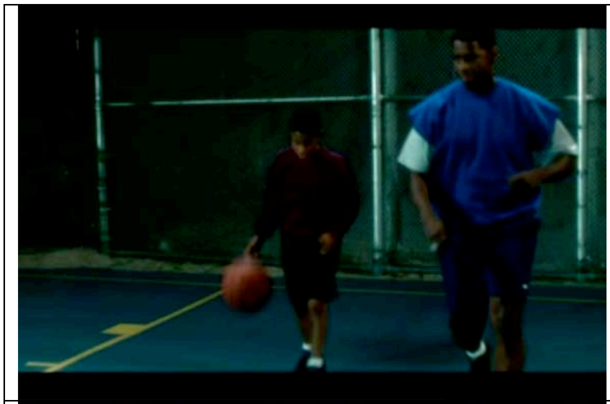	*He Got Game*. Dir. Spike Lee. Touchstone Pictures: 1998.	A boy dribbles a basketball while a man walks next to him.
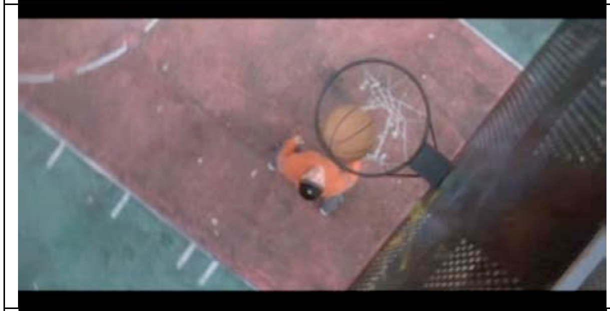	*The Art of War*. Dir. Christian Duguay. Warner Bros: 2000.	A series of tight and aerial camera shots follows a basketball.
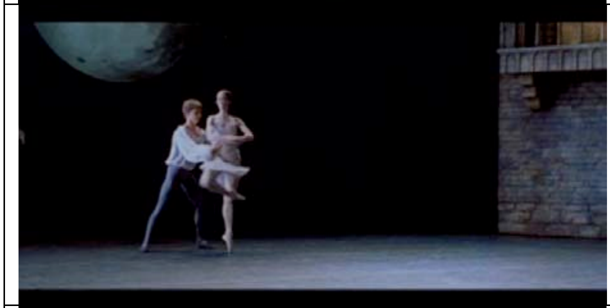	*Center Stage*. Dir. Nicholas Hytner. Columbia Pictures: 2001.	A man holds a woman as she twirls around on point.
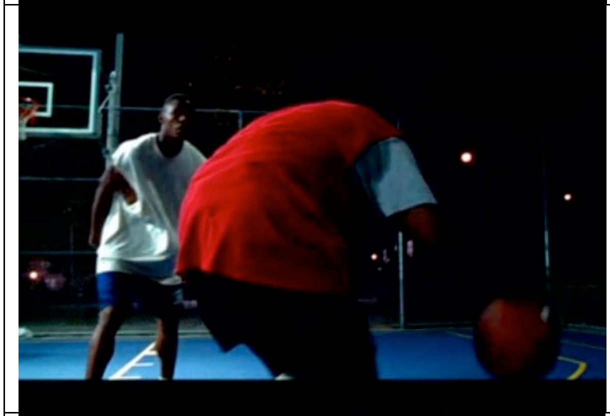	*He Got Game*. Dir. Spike Lee. Touchstone Pictures: 1998.	Man dribbles a basketball while another tries to block him.
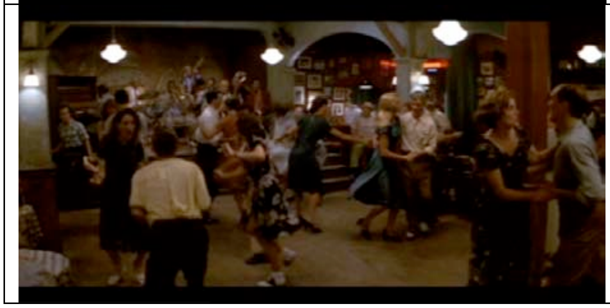	*A League of Their Own*. Dir. Penny Marshall. Columbia Pictures: 1992.	A crowd of people dances.

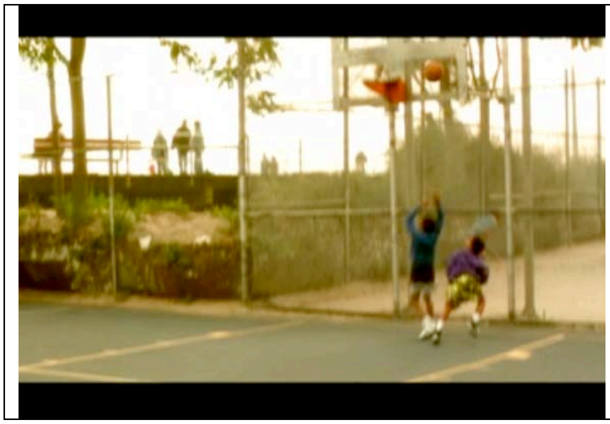	*He Got Game*. Dir. Spike Lee. Touchstone Pictures: 1998.	Two children play basketball; one attempts to block the other as he throws the ball toward the net.
