# Supplementary material for: Repeated Exposure to Media Violence Is Associated with Diminished Response in an Inhibitory Frontolimbic Network
Source: PLoS One. 2007 Dec 5;2(12):e1268. doi: 10.1371/journal.pone.0001268 (PMC2092389; doi:10.1371/journal.pone.0001268)
Supplement: Appendix S2 — This file contains additional information about the eye-tracking analysis. (0.04 MB PDF) [file pone.0001268.s009.pdf]

Ten additional subjects watched the video stimuli (under the same conditions as the scanned subjects) while we recorded gaze position. Using repeated measures ANOVA, we separately analyzed (1) distance traveled by each subject's gaze and (2) number of saccades, using stimulus category and prior exposure as fixed effects, as well as subject number as a random effect. Each subject contributed two values for each stimulus: total number of saccades and total distance traveled, both normalized by stimulus duration.

Stimulus category had a significant effect on mean number of saccades ($p<0.0001$) but not mean gaze distance traveled, while prior experience had no significant effect on either ($p=0.78$ and $p=0.60$, respectively). Post-hoc multiple comparisons of these data ($\alpha=0.05$, Bonferroni correction) revealed that saccades were fewer during the fearful condition than during both the violent and neutral conditions, which did not differ.

These data reveal several important facts. First, for both of these measures, either one or both of the control conditions did not differ from the violent condition. Second, the presence of a difference between the violent/neutral and fearful conditions in total number of saccades, but not in mean distance traveled, may relate to the fact that the contents of the fearful clips were not moving as quickly as in the violent and neutral conditions, in which there was significant physical activity. Subjects thus likely used slower, smooth pursuits to track the figures during the fearful condition. These analyses suggest that the fearful and neutral conditions are suitable controls for the violent condition.
