# Supplementary material for: Repeated Exposure to Media Violence Is Associated with Diminished Response in an Inhibitory Frontolimbic Network
Source: PLoS One. 2007 Dec 5;2(12):e1268. doi: 10.1371/journal.pone.0001268 (PMC2092389; doi:10.1371/journal.pone.0001268)
Supplement: Appendix S3 — This file contains the modified aggression questionnaire. (0.04 MB PDF) [file pone.0001268.s010.pdf]

1	Some of my friends think I am a hothead
2	I have become so mad that I have broken things
3	I can't help getting into arguments when people disagree with me
4	Once in a while, I can't control the urge to strike another person
5*	I am an even-tempered person
6	I flare up quickly but get over it quickly
7	Given enough provocation, I may hit another person
8	When people annoy me, I may tell them what I think of them
9*	I can think of no good reason for ever hitting a person
10	I have trouble controlling my temper
11	When frustrated, I let my irritation show
12	If somebody hits me, I hit back
13	I sometimes feel like a powder keg ready to explode
14	There are people who pushed me so far that we came to blows
15	My friends say that I'm somewhat argumentative
16	Sometimes I fly off the handle for no good reason
17	I get into fights a little more than the average person

Each item was rated along the following axis:
1: extremely uncharacteristic of me
5: extremely characteristic of me

* = reverse-scored
All of the questions received equal weight in computing total score.

For details on each subject's responses to these questions, as well as the variance generated by each question, please supplementary table 1.
